# Non-autonomous multi-rogue waves for spin-1 coupled nonlinear Gross-Pitaevskii equation and management by external potentials

**DOI:** 10.1038/s41598-017-10205-4

**Published:** 2017-09-06

**Authors:** Li Li, Fajun Yu

**Affiliations:** School of Mathematics and Systematic Sciences, Shenyang Normal University, Shenyang, 110034 China

## Abstract

We investigate non-autonomous multi-rogue wave solutions in a three-component(spin-1) coupled nonlinear Gross-Pitaevskii(GP) equation with varying dispersions, higher nonlinearities, gain/loss and external potentials. The similarity transformation allows us to relate certain class of multi-rogue wave solutions of the spin-1 coupled nonlinear GP equation to the solutions of integrable coupled nonlinear Schrödinger(CNLS) equation. We study the effect of time-dependent quadratic potential on the profile and dynamic of non-autonomous rogue waves. With certain requirement on the backgrounds, some non-autonomous multi-rogue wave solutions exhibit the different shapes with two peaks and dip in bright-dark rogue waves. Then, the managements with external potential and dynamic behaviors of these solutions are investigated analytically. The results could be of interest in such diverse fields as Bose-Einstein condensates, nonlinear fibers and super-fluids.

## Introduction

Rogue waves(RWs) are called the monster waves or extreme waves in the ocean, which are catastrophic natural physical phenomena (thunderstorms, earthquakes and hurricanes)^1–5^. The character of the rogue wave is: the amplitudes much higher than the average wave crests around them^2^. The nonlinear Schrödinger(NLS) equation and its variants are the most important basic models of mathematical physics in physical and engineering sciences. Especially some important applications are presented in nonlinear optics^6^ and water wave tanks^7^. It universality stimulates a great deal of attention devoted to searching for exact solutions of the generalized NLS models. The studies of rogue wave in single-component system have indicated that the rational solution of the NLS equation can be used to describe the phenomenon well^8, 9^. Ohta and Yang consdier the dynamics of rogue waves in the Davey-Stewartson Eq. 4. Searching for the rogue waves or rational solutions of NLS equation is an interesting work, which can describe new physical phenomena.

The spinor Bose-Einstein condensates (BECs), which have been experimentally realized in optical potentials, and exhibit a rich variety of magnetic effects^10–12^. For example, the spin-exchange interaction can be tuned by optics^13, 14^ or microwave Feshbach resonance techniques^15^ in a spinor condensate. The sign of the interaction in the ground state of spin-1 bosons is important, it gives rise to the phenomena are not be presented in the single component BECs^16–18^. The spinor BECs have been realized in the optical traps, the direction of the spin can change dynamically due to collisions between the atoms^19–21^. The spinor BECs can exhibit a rich variety of magnetic phenomena, including magnetic crystallization, spin textures and fractional vortices^22–24^.

A variety of complex systems, such as BECs, nonlinear optical fibers, etc., usually involve more than one component^25^. Recently, some problems are extended to RWs in two-component systems^25–28^, and some new structures(bright-dark RWs) have been presented numerically^27^ and analytically^28^. Moreover, it is found that two RWs can emerge in the coupled system which is quite distinct from the high-order RWs in a one-component system^28^. In the two-component system, the interaction between a RW and other nonlinear waves is also a hot topic^27, 28^. The various approximations are also employed to study the solitons such as bright and dark solitons in the *F* = 1 spinor BECs in ref. 25.

On the other hand, some methods are proposed to solve the nonlinear partial differential equations (PDEs) in refs 29–31, e.g., the Darboux transformation(DT)^32^, inverse scattering transformation^33^, B*ä*cklund transformation (BT)^34, 35^, Painlevé test^36^ and Hirota method^37^. Among those methods, the BT can also be used to obtain a nontrivial solution from a seed solution in refs 37, 38. In recently, some important scientific studies of rational solutions and lump solutions in soliton equations are derived. An explicit formulation of a 2n-dimensional Lax integrable system is established with Darboux transformation by Ma in ref. 39, which contains some rational and analytic solutions. In ref. 40, Ma and You present a recursive procedure for constructing rational solutions of the Toda lattice equation through the Casoratian formulation, and obtain directly a broad class of rational solutions. Three ansätze transformations are used to construct exact solutions of NLS equation in ref. 41, and a more generalized rational solution including Ma soliton is presented. Ma, Zhou and Dougherty study systematically the lump-type solutions of nonlinear differential equations based on bilinear forms or generalized bilinear forms in ref. 42. The DT is a powerful method to construct the soliton solutions for the integrable equations. There are different methods to derive the DT, for instance the operator decomposition method, gauge transformation, loop group method and Riemann-Hilbert method. The DT can be used to construct multi-soliton and coherent structure solutions of nonlinear integrable equations in both (1 + 1) and (2 + 1) dimensions. Matveev and Salle consider some important applications on DT in solitons, and give rise to the transformation properties of the 1-dimensional NLS equation in ref. 43. In ref. 44, the Darboux invariance of differential-difference evolution equation is defined and proved, some formulas involving determinants are applicable to an arbitrary initial solution of the Toda equation. Some applications of the DT method to study the reduced Maxwell-Bloch system and the self-induced transparency equations are considered, which systems describe the propagations of ultrashort opticas in ref. 45. The binary DT and the Zakharov-Shabat dressing method are considered for the Toda lattice equation by Babich, Matveev and Sail in ref. 46.

The rational dressing method was originally proposed in refs 47, 48 and developed in ref. 49. This method enables one to construct multi-soliton solutions and analyse soliton interactions in detail using basic knowledge of Linear Algebra. And, it allows one to apply the dressing method of Zakharov-Shabat-Mikhailov^47–49^ for calculating their soliton solutions. Recently, Mikhailov *et al*. develop the dressing method to study the exact solutions for the vector sine-Gordon equation^50^ and two-dimensional periodic Volterra system^51^. Some formulas for position shift of the kink and phase shifts of the breather are given. Especially, Mikhailov *et al*. propose a method for construction of Darboux transformations in ref. 52, which is a new development of the dressing method for Lax operators invariant under a reduction group. And they derive new vector Yang-Baxter map and integrable discrete vector sine-Gordon equation on a sphere.

In this work, extending the ideas in refs 38, 53–55, we further investigate a three-component coupled system with higher-order terms. The non-autonomous multi-rogue waves of the three-component coupled GP equation are reported by using the similarity transformation and Darboux transformation. Some structures with two peaks in bright rogue wave and dark rogue wave are found in the coupled system, which are quite distinct from the well-known shapes one presented before. Furthermore, we construct some new explicit solutions, which can be used to describe the dynamics of non-autonomous RWs, and discuss the managements by external potentials in BECs and nonlinear optics.

## Results

### Similarity reduction for the three-component coupled GP equation

It is well-known that the coupled GP equation is often used to describe the interactions among the modes in nonlinear optics, components in BEC, etc. We consider the three-component coupled GP equation with variable coefficients, which can be written as following

1a $$\begin{array}{rcl}i{{\rm{\Psi }}}_{\pm 1t} & = & -{{\rm{\Psi }}}_{\pm 1xx}+{V}_{\pm 1}(x,t){{\rm{\Psi }}}_{\pm 1}+i{{\rm{\Gamma }}}_{\pm 1}(t){{\rm{\Psi }}}_{\pm 1}+[({c}_{0}+{c}_{2}){A}_{\pm 1}(t){|{{\rm{\Psi }}}_{\pm 1}|}^{2}\\  &  & +\,({c}_{0}+{c}_{2}){B}_{\pm 1}(t){|{{\rm{\Psi }}}_{0}|}^{2}]{{\rm{\Psi }}}_{\pm 1}\\  &  & +\,({c}_{0}-{c}_{2}){C}_{\pm 1}(t){|{{\rm{\Psi }}}_{\mp 1}|}^{2}{{\rm{\Psi }}}_{\pm 1}+{c}_{2}{D}_{\pm 1}(t){{\rm{\Psi }}}_{\mp 1}^{\ast }{{\rm{\Psi }}}_{0}^{2},\end{array}$$

1b $$\begin{array}{rcl}i{{\rm{\Psi }}}_{0t} & = & -{{\rm{\Psi }}}_{0xx}+{V}_{0}(x,t){{\rm{\Psi }}}_{0}+i{{\rm{\Gamma }}}_{0}(t){{\rm{\Psi }}}_{0}+[({c}_{0}+{c}_{2}){A}_{0}(t){|{{\rm{\Psi }}}_{+1}|}^{2}\\  &  & +\,({c}_{0}+{c}_{2}){C}_{0}(t){|{{\rm{\Psi }}}_{-1}|}^{2}]{{\rm{\Psi }}}_{0}\\  &  & +\,{c}_{0}{B}_{0}(t){|{{\rm{\Psi }}}_{0}|}^{2}{{\rm{\Psi }}}_{0}+2{c}_{2}{D}_{0}(t){{\rm{\Psi }}}_{0}^{\ast }{{\rm{\Psi }}}_{+1}{{\rm{\Psi }}}_{-1},\end{array}$$

where Ψ_*j*_ = Ψ_*j*_(*x*, *t*)(*j* = +1, 0, −1), ∂ = ∂/∂*x*, *x* is the propagation variable and *t* is the transverse variable. The *V*_*j*_(*t*) and *D*_*j*_(*t*)(*j* = +1, 0, −1) represent the group-velocity dispersion and the third-order dispersion, respectively. The parameters *A*_*j*_(*t*) and *B*_*j*_(*t*) are related to the self-steepenings, *C*_*j*_(*t*) is the nonlinearity parameter, and the gain/loss coefficient Γ_*j*_(*t*) is real-valued function of time. One is the nonlinear density-density interaction which is associated with the *c*_0_ term, while the other is the spin-exchange coupling between the hyper fine states, which is associated with the *c*_2_ term.

We search for a similarity transformation connecting solutions of Eq. (1) with the 3-component coupled nonlinear schrödinger(CNLS) equation. The CNLS equation in a dimensionless form^56, 57^ is given as following

2a $$i{{\rm{\Phi }}}_{\pm 1\tau }=-{{\rm{\Phi }}}_{\pm 1\eta \eta }+({c}_{0}+{c}_{2})[{|{{\rm{\Phi }}}_{\pm 1}|}^{2}+{|{{\rm{\Phi }}}_{0}|}^{2}]{{\rm{\Phi }}}_{\pm 1}+({c}_{0}-{c}_{2}){|{{\rm{\Phi }}}_{\mp 1}|}^{2}{{\rm{\Phi }}}_{\pm 1}+{c}_{2}{{\rm{\Phi }}}_{\mp 1}^{\ast }{{\rm{\Phi }}}_{0}^{2},$$

2b $$i{{\rm{\Phi }}}_{0\tau }=-{{\rm{\Phi }}}_{0\eta \eta }+({c}_{0}+{c}_{2})[{|{{\rm{\Psi }}}_{+1}|}^{2}+{|{{\rm{\Phi }}}_{-1}|}^{2}]{{\rm{\Phi }}}_{0}+{c}_{0}{|{{\rm{\Phi }}}_{0}|}^{2}{{\rm{\Phi }}}_{0}+2{c}_{2}{{\rm{\Phi }}}_{0}^{\ast }{{\rm{\Phi }}}_{+1}{{\rm{\Phi }}}_{-1},$$

the physical fields Φ_*i*_(*η*, *τ*)(*i* = ±1, 0) are functions of the variables *η* = *η*(*x*, *t*) and *τ* = *τ*(*t*). The *c*_0_ and *c*_2_ are the mean-field and the spin-exchange interaction, and ${\bar{c}}_{2}=4\pi {h}^{2}({a}_{2}-{a}_{0}\mathrm{)/(3}M)$ is the spin-exchange interaction, which is ferromagnetic if ${\bar{c}}_{2} < 0$ (as ^87^Rb) and anti-ferromagnetic if ${\bar{c}}_{2} > 0$ (as ^23^Na). The energy is in units of $|{\bar{c}}_{n}|$ with n being the average particle density. Here a_0_ and a_2_ denote the s-wave scattering lengths of the total spin-0 and spin-2 channels, respectively.

It has been reported that solitons could collide inelastically, and there are shape-changing collisions for a coupled system, which is different from an uncoupled system^13^. However, it is not possible to study a vector RWs with a trivial background, we will solve Eq. (2) with the nontrivial seed solutions

According to the ansatz methods^38, 53, 54, 58–60^, we search for the solutions of physical fields

3 $${{\rm{\Psi }}}_{m}(x,t)={\rho }_{m}(t){e}^{i{\phi }_{m}(x,t)}{{\rm{\Phi }}}_{m}(\eta (x,t),\tau (t)),m=+1,0,-1,$$

with *ρ*(*t*) and *φ*(*x*, *t*) are the real-value functions of the indicated variables. The ansatz(3) can construct the relations between Eq. (1) and Eq. (2). And, we substitute the transformation (3) into Eq. (1) and get the following systems:

4a $${\eta }_{xx}=0,{\eta }_{t}+{\phi }_{mx}{\eta }_{x}=0,$$

4b $${\rho }_{mt}+{\rho }_{m}{\phi }_{xx}-{\rho }_{m}{{\rm{\Gamma }}}_{m}=0,$$

4c $${\tau }_{t}={({\eta }_{x})}^{2},{V}_{m}(x,t)-{\phi }_{mt}-\frac{1}{2}{\phi }_{mx}^{2}=0,$$

4d $${A}_{m}(t)=\frac{{\tau }_{t}(t)}{{\rho }_{+1}^{2}(t)},{B}_{m}(t)=\frac{{\tau }_{t}(t)}{{\rho }_{0}^{2}(t)},$$

4e $${C}_{m}(t)=\frac{{\tau }_{t}(t)}{{\rho }_{-1}^{2}(t)},{D}_{\pm 1}(t)=\frac{{\tau }_{t}(t){\rho }_{\pm 1}(t)}{{\rho }_{0}^{2}(t){\rho }_{\mp 1}(t)},$$

4f $${D}_{0}(t)=\frac{{\tau }_{t}(t)}{{\rho }_{+1}(t){\rho }_{-1}(t)}\mathrm{.}$$

We solve *η*_*xx*_ = 0, and obtain the functions *η*(*x*, *t*), *φ*(*x*, *t*) and *ρ*_*m*_(*t*). If the function *τ*(*t*) is given, the functions *A*_*m*_(*t*), *B*_*m*_(*t*), *C*_*m*_(*t*) and *D*_*m*_(*t*) can be expressed. Thus, we can establish a correspondence between selected solutions of Eq. (1) and CNLS Eq. (2). In particular, we can obtain some non-autonomous multi-rogue wave solutions of Eq. (1).

Solving Eq. (4a), we get the similarity variables *η*(*x*, *t*) and *τ*(*t*) in the forms

5 $$\eta (x,t)=\alpha (t)x+ab(t)+c,$$

and

6 $$\tau (t)={\int }_{0}^{t}{\alpha }^{2}(s)ds,$$

and obtain the function *ρ*_*m*_(*t*), the phase *φ*(*x*, *t*) and external potential *V*_*m*_(*x*, *t*) in the following forms

7a $${\rho }_{m}(t)={\rho }_{0}{e}^{{\int }_{0}^{t}{\rm{\Gamma }}(s)ds},$$

7b $${\phi }_{m}(x,t)=-\frac{1}{2}{(\mathrm{ln}\alpha (t))}_{t}{x}^{2}-\frac{a{b}_{t}(t)}{\alpha (t)}x+d,$$

7c $${V}_{m}(x,t)=-\frac{1}{2}{(\mathrm{ln}\alpha (t))}_{tt}{x}^{2}-{(\frac{a{b}_{t}(t)}{\alpha (t)})}_{t}x+\frac{1}{2}{(-{(\mathrm{ln}\alpha (t))}_{t}x-\frac{a{b}_{t}(t)}{\alpha (t)})}^{2},$$

where the *d* and *ρ*_0_ are constants.

We substitute Eqs (5, 6) and (7) into the ansatz (3) and prove the following result:

8 $${{\rm{\Psi }}}_{m}(x,t)={\rho }_{m0}{{\rm{\Phi }}}_{m}(\alpha (t)x+ab(t))+c,{\int }_{0}^{t}{\alpha }^{2}(s)ds){e}^{{\int }_{0}^{t}{\rm{\Gamma }}(s)ds+i(-\frac{1}{2}{(\mathrm{ln}\alpha (t))}_{t}{x}^{2}-\frac{a{b}_{t}(t)}{\alpha (t)}x+d)}\mathrm{.}$$

**Figure 1:**
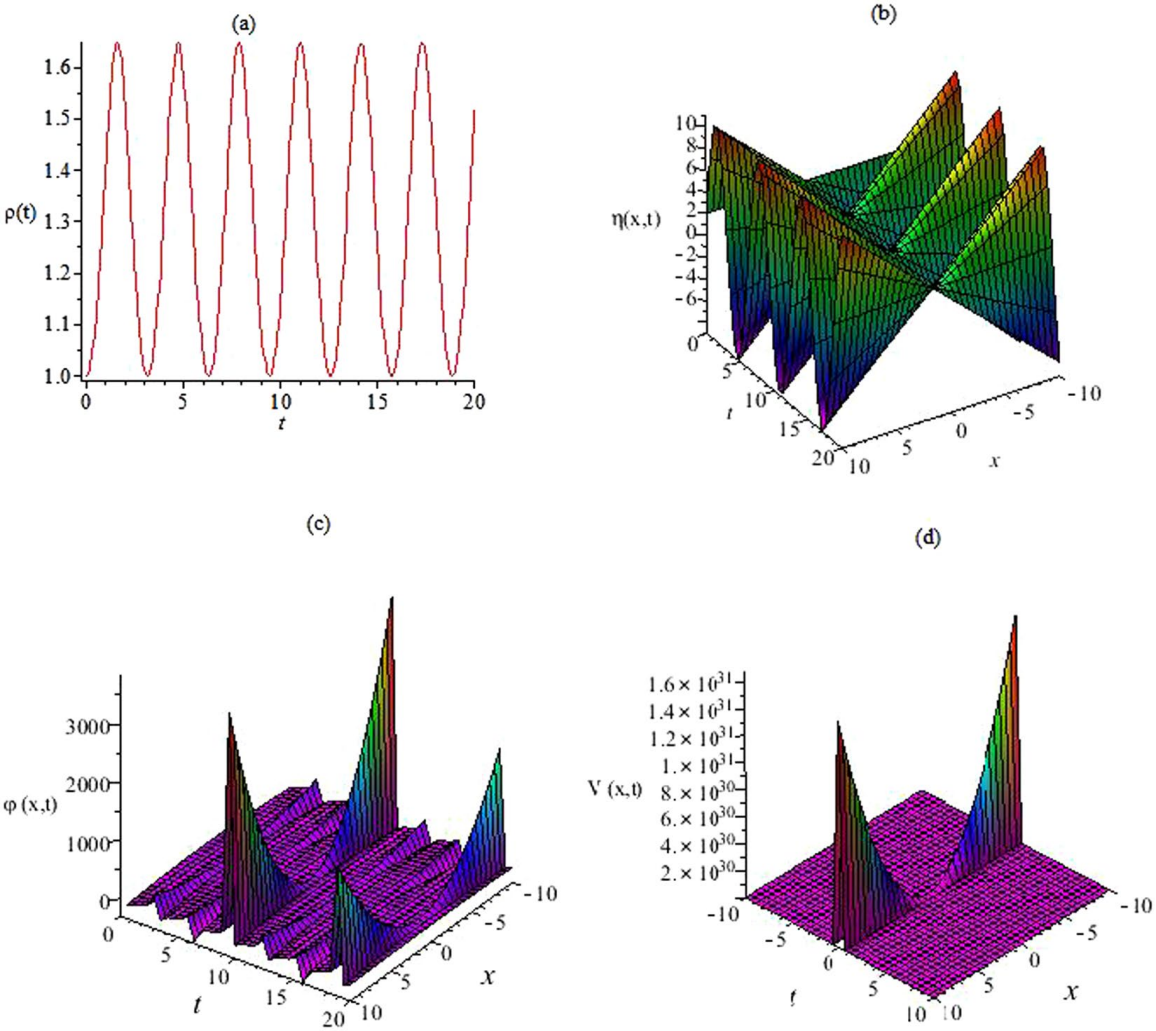
The functions *ρ*(*t*), *η*(*x*, *t*), *φ*(*x*, *t*) and external potential *V*(*x*, *t*). (**a**) The nonlinearity *ρ*(*t*) is given by Eq. (7) with Γ(*t*) = sin(*t*)cos(*t*), *ρ*_0_ = 1. (**b**) The *η*(*x*, *t*) is given by Eq. (5) with *α*(*t*) = sin(*t*), *b*(*t*) = cos(*t*), *a* = 1, *c* = 1. (**c**) The function *φ*(*x*, *t*) is given by Eq. (7) with *α*(*t*) = sin(*t*), *b*(*t*) = cos(*t*), *d* = 1. (**d**) The external potential *V*(*x*, *t*) is given by Eq. (7) with *α*(*t*) = sin(*t*), *b*(*t*) = cos(*t*).

**Figure 2:**
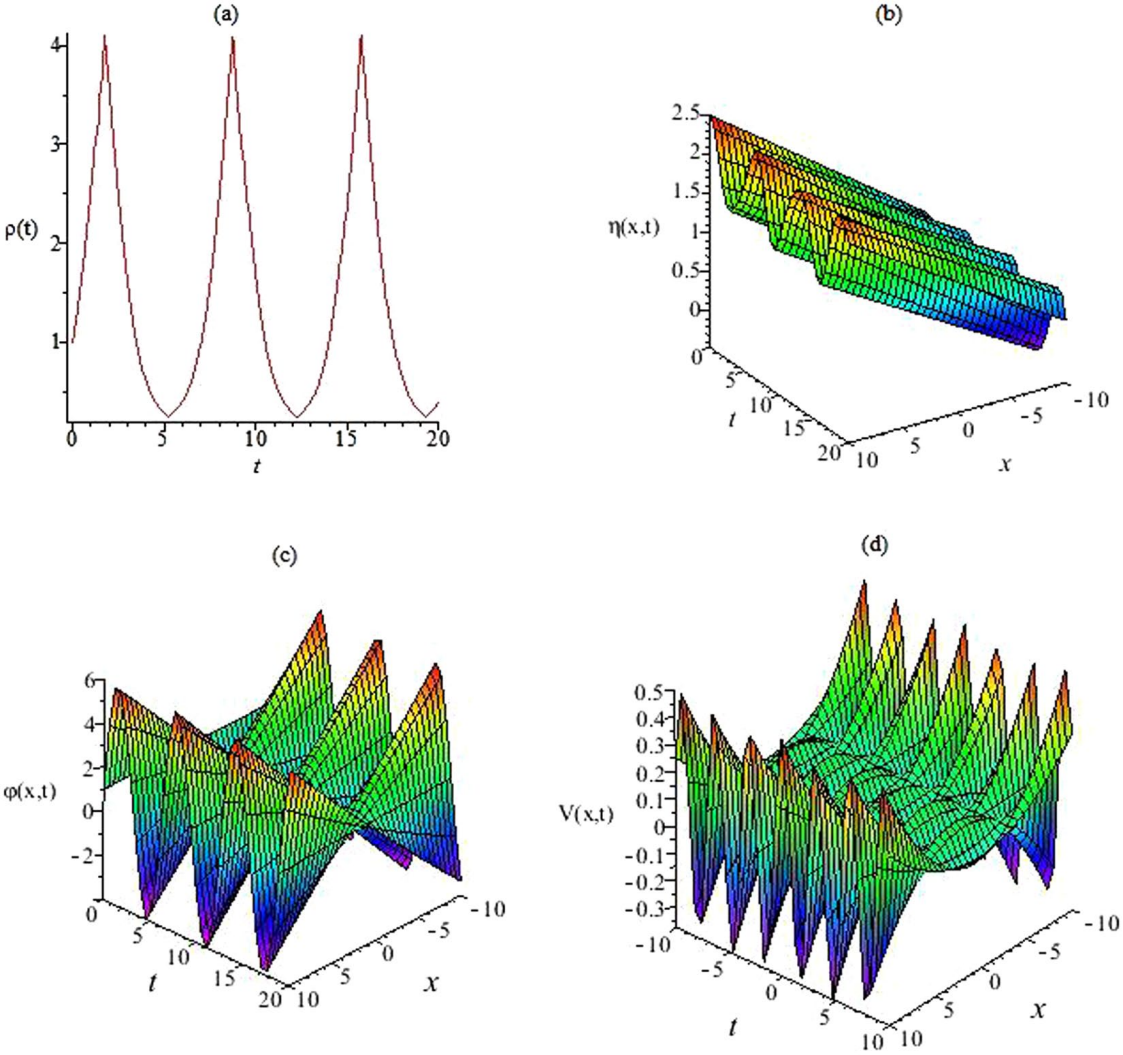
The functions *ρ*(*t*), *η*(*x*, *t*), *φ*(*x*, *t*) and external potential *V*(*x*, *t*). (**a**) The nonlinearity *ρ*(*t*) is given by Eq. (7) with Γ(*t*) = *dn*(*t*, *k*), *ρ*_0_ = 1, *k* = 0.6. (**b**) The *η*(*x*, *t*) is given by Eq. (5) with *α*(*t*) = 0.1*dn*(*t*, *k*), *b*(*t*) = 0.5cos(*t*), *a* = 1, *c* = 1, *k* = 0.6. (**c**) The function *φ*(*x*, *t*) is given by Eq. (7) with *α*(*t*) = 0.1*dn*(*t*, *k*), *b*(*t*) = 0.5cos(*t*), *d* = 1, *k* = 0.6. (**d**) The external potential *V*(*x*, *t*) is given by Eq. (7) with *α*(*t*) = 0.1*dn*(*t*, *k*), *b*(*t*) = 0.5cos(*t*), *k* = 0.6.

We choose the different parameters *ρ*_*m*0_, *a*, *c*, *d* and the different functions *α*(*t*), *γ*(*t*), *b*(*t*), the Figs 1 and 2 depict the profiles of *ρ*(*t*), *η*(*x*, *t*), *φ*(*x*, *t*) and the external potential *V*(*x*, *t*) in Eqs (5) and (7). We present the quadratic external potential in Fig. 2(d).

### New rational solutions with arbitrary constants for CNLS equation

The rational solutions are considered in many works, such as the “Ma solitons” (MS) are presented in ref. 6, the “Akhmediev breathers” (ABs) are found in refs 7–9. Thus, the studies of rational solutions are of fundamental importance. They may resolve the mystery of rogue waves in the ocean and help in creating useful rogue waves in optical fibers. We take the generalized Darboux transformation(GDT) technique to construct the new rational solutions with arbitrary constants for the CNLS Eq. (2).

We consider the system with the coupling constants *c*_0_ = *c*_2_ = −*c* < 0, Φ → (Φ_+1_, $\sqrt{{\rm{2}}}{{\rm{\Phi }}}_{0}$, Φ_−1_)^T^, Eq. (2) becomes as following

9 $$i{\partial }_{\tau }{\bf{Q}}+{\partial }_{\eta }^{2}{\bf{Q}}+2{\bf{Q}}{Q}^{+}{\bf{Q}}=0,$$

where $${\bf{Q}}=(\begin{array}{cc}{{\rm{\Phi }}}_{+1} & {{\rm{\Phi }}}_{0}\\ {{\rm{\Phi }}}_{0} & {{\rm{\Phi }}}_{-1}\end{array})$$ and the “+” denotes the conjugate transpose, Eq. (9) is a completely integrable system. In ref. 61, a soliton hierarchy of multicomponent NLS equation is generated from an arbitrary order matrix spectral problem, which has a multicomponent Lax pairs. In this section, we consider the 3-component Lax pairs, which is similar to the multicomponent Lax pairs with *m* = 3 in ref. 61. We here aim to develop the GDT for Eq. (9) with the respective Lax pairs.

The Lax pairs **U** and **V** are presented as following

10 $${\bf{U}}=\lambda {\bf{J}}+{\bf{P}},\quad {\bf{V}}=2i{\lambda }^{2}{\bf{J}}+2i\lambda {\bf{P}}+i{\bf{W}},$$

with

$${\bf{J}}=(\begin{array}{cc}{\bf{I}} & {\bf{O}}\\ {\bf{O}} & -\,{\bf{I}}\end{array}),{\bf{P}}=(\begin{array}{cc}{\bf{O}} & {\bf{Q}}\\ -{\bf{Q}} & {\bf{O}}\end{array}),{\bf{W}}=(\begin{array}{cc}{\bf{Q}}{{\bf{Q}}}^{+} & {{\bf{Q}}}_{x}\\ {{\bf{Q}}}_{x}^{+} & -{{\bf{Q}}}^{+}{\bf{Q}}\end{array}),$$

here **I** denotes the 2 × 2 unit matrix, **O** is the 2 × 2 zero matrix,

11 $${\boldsymbol{\Psi }}=(\begin{array}{c}{{\rm{\Psi }}}_{1}\\ {{\rm{\Psi }}}_{2}\end{array}),{{\rm{\Psi }}}_{1}=(\begin{array}{cc}{r}_{11} & {r}_{12}\\ {r}_{21} & {r}_{22}\end{array}),{{\rm{\Psi }}}_{2}=(\begin{array}{cc}{s}_{11} & {s}_{12}\\ {s}_{21} & {s}_{22}\end{array}),$$

with dependent variables *r*_*pq*_(*η*, *τ*), *s*_*pq*_(*η*, *τ*)(*p*, *q* = 1, 2) and λ is the spectral parameter.

Considering a linear system as following:

12 $${{\boldsymbol{\Psi }}}_{\eta }={\rm{U}}{\boldsymbol{\Psi }},{{\boldsymbol{\Psi }}}_{\tau }={\rm{V}}{\boldsymbol{\Psi }},$$

from the system (12) and the condition of compatibility

$${{\boldsymbol{\Psi }}}_{\eta \tau }={{\boldsymbol{\Psi }}}_{\tau \eta },$$

we can get the CNLS Eq. (2).

Based on the Lax pairs (12) and introducing a transformation in the form

13 $$\tilde{{\boldsymbol{\Psi }}}=(\lambda -{\bf{S}}){\boldsymbol{\Psi }},\,\,{\bf{S}}={\bf{D}}{\rm{\Lambda }}{{\bf{D}}}^{-1},\,\,{\rm{\Lambda }}=(\begin{array}{cc}{\lambda }_{1}{\bf{I}} & {\bf{O}}\\ {\bf{O}} & {\lambda }_{2}{\bf{I}}\end{array}),$$

where **D** is a nonsingular matrix that satisfies **D**_*x*_ = **JD**Λ + **PD**, and letting

14 $${\tilde{{\boldsymbol{\Psi }}}}_{x}=\tilde{{\bf{U}}}\tilde{{\boldsymbol{\Psi }}},\quad \tilde{{\bf{U}}}=\lambda {\bf{J}}+{{\bf{P}}}_{1},\quad {{\bf{P}}}_{1}=(\begin{array}{cc}{\bf{O}} & {{\bf{Q}}}_{1}\\ -{{\bf{Q}}}_{1}^{+} & {\bf{O}}\end{array}),$$

one obtains

15 $${{\bf{P}}}_{1}={\bf{P}}+{\bf{J}}{\bf{S}}-{\bf{S}}{\bf{J}}\mathrm{.}$$

Also, one can verify the following involution property of the above linear equations: if

$${\boldsymbol{\Psi }}=(\begin{array}{c}{{\rm{\Psi }}}_{1}\\ {{\rm{\Psi }}}_{2}\end{array})$$

is an eigenfunction corresponding to λ, where Ψ_*j*_(*j* = 1, 2) are 2 × 2 matrices, then

$$(\begin{array}{c}-{{\rm{\Psi }}}_{2}^{\ast }\\ {{\rm{\Psi }}}_{1}^{\ast }\end{array})$$

is an eigenfunction corresponding to −λ^*^, where we have used the fact that **Q** ^*^ = **Q** ^+^, and **Q** is symmetric. Thus we can take **D** in the form

$${\bf{D}}=(\begin{array}{cc}{{\rm{\Psi }}}_{1} & -{{\rm{\Psi }}}_{2}^{\ast }\\ {{\rm{\Psi }}}_{2} & {{\rm{\Psi }}}_{1}^{\ast }\end{array}),\quad {\rm{\Lambda }}=(\begin{array}{cc}\lambda {\bf{I}} & {\bf{O}}\\ {\bf{O}} & -{\lambda }^{\ast }{\bf{I}}\end{array}),$$

to obtain

$${\bf{S}}=\lambda (\begin{array}{cc}{\bf{I}} & {\bf{O}}\\ {\bf{O}} & {\bf{I}}\end{array})+(\lambda +{\lambda }^{\ast })(\begin{array}{cc}-{{\bf{S}}}_{11} & {{\bf{S}}}_{12}\\ {{\bf{S}}}_{21} & -{{\bf{S}}}_{22}\end{array}),$$

where the matrix elements of **S** are given by ${{\bf{S}}}_{11}={({\bf{I}}+{{\rm{\Psi }}}_{1}{{\rm{\Psi }}}_{2}^{-1}{{\rm{\Psi }}}_{1}^{\ast }{{\rm{\Psi }}}_{2}^{\ast -1})}^{-1}$, ${{\bf{S}}}_{12}={({{\rm{\Psi }}}_{2}{{\rm{\Psi }}}_{1}^{-1}+{{\rm{\Psi }}}_{1}^{\ast }{{\rm{\Psi }}}_{2}^{\ast -1})}^{-1}$, ${{\bf{S}}}_{21}=$ ${({{\rm{\Psi }}}_{2}^{\ast }{{\rm{\Psi }}}_{1}^{\ast -1}+{{\rm{\Psi }}}_{1}{{\rm{\Psi }}}_{2}^{-1})}^{-1}$ and ${{\bf{S}}}_{22}={({\rm{I}}+{{\rm{\Psi }}}_{2}{{\rm{\Psi }}}_{1}^{-1}{{\rm{\Psi }}}_{2}^{\ast }{{\rm{\Psi }}}_{1}^{\ast -1})}^{-1}$.

The Darboux transformation for Eq. (9) takes from **P**_1_ as following

16 $${{\bf{Q}}}_{1}={\bf{Q}}+\mathrm{2(}\lambda +{\lambda }^{\ast })({{\rm{\Psi }}}_{2}{{\rm{\Psi }}}_{1}^{-1}+{{\rm{\Psi }}}_{1}^{\ast }{{\rm{\Psi }}}_{2}^{\ast -1}{)}^{-1},$$

it should be noted that **S** ^*^ = **S** implies the above-mentioned property ${{\bf{Q}}}_{1}^{\ast }={{\bf{Q}}}_{1}^{+}$. From Eqs (12) and (16), it can be deduced that Eq. (16) generates a new solution **Q**_1_ for Eq. (9) once a seed solution **Q** is known. In particular, a one-soliton solution can be generated if the seed is a trivial (zero) state. If, taking **Q**_1_ as the new seed solution, one can derive from Eq. (16) the corresponding two-soliton solution. This can be continued as a recursion procedure generating multi-soliton solutions.

According to Eqs (11) and (12), the following large set of linear equations is presented, which contains 16 linear equations of *r*_*pq*_(*η*, *τ*), *s*_*pq*_(*η*, *τ*)(*p*, *q* = 1, 2) as following

17 $${(\begin{array}{ll}{r}_{11} & {r}_{12}\\ {r}_{21} & {r}_{22}\\ {s}_{11} & {s}_{12}\\ {s}_{21} & {s}_{22}\end{array})}_{\eta }={\bf{U}}(\begin{array}{ll}{r}_{11} & {r}_{12}\\ {r}_{21} & {r}_{22}\\ {s}_{11} & {s}_{12}\\ {s}_{21} & {s}_{22}\end{array}),$$

and

18 $${(\begin{array}{ll}{r}_{11} & {r}_{12}\\ {r}_{21} & {r}_{22}\\ {s}_{11} & {s}_{12}\\ {s}_{21} & {s}_{22}\end{array})}_{\tau }={\bf{V}}(\begin{array}{ll}{r}_{11} & {r}_{12}\\ {r}_{21} & {r}_{22}\\ {s}_{11} & {s}_{12}\\ {s}_{21} & {s}_{22}\end{array}),$$

where the 16 linear equations in Eqs (17) and (18) are given in the Appendix A of Supplementary information, and ${\varphi }_{10}={\varphi }_{0}^{\ast },\,{\varphi }_{-11}={\varphi }_{-1}^{\ast },\,{\varphi }_{11}={\varphi }_{1}^{\ast }$. Eqs (12, 17) and (18) establish a one-to-one correspondence between the solution $$(\begin{array}{c}{{\rm{\Psi }}}_{1}\\ {{\rm{\Psi }}}_{2}\end{array})$$ of the CNLS equation and the solution of the linear system (12).

Noting that **Q**_1_ in Darboux transformation Eq. (16) should be a symmetric matrix, we require

19 $${r}_{21}={r}_{12},{s}_{21}={s}_{12}.$$

Inserting Eq. (16) into the linear system of Eq. (12), we obtain the following compatible conditions:

20 $${r}_{21}=\frac{{r}_{11}-{r}_{22}}{2},{s}_{21}=\frac{{s}_{11}-{s}_{22}}{2}.$$

We choose a “seed solution” $${Q}_{0}=(\begin{array}{cc}1 & 1\\ 1 & -1\end{array}){e}^{4i\tau }$$ and use the GDT to obtain the higher order rational solutions for the 3-component CNLS equation.

Our next step is to find two linear functions *r*_*ij*_(*η*, *τ*) and *s*_*ij*_(*η*, *τ*) that make system (12) compatible with **Q** = **Q**_0_. The GDT scheme gives rise to

21 $${{\bf{Q}}}_{0}\to ({r}_{11}[1],{r}_{22}[1],{s}_{11}[1],{s}_{22}[1])\to {{\bf{Q}}}_{1}\to ({r}_{11}[2],{r}_{22}[2],{s}_{11}[2],{s}_{22}[2])\to {{\bf{Q}}}_{2}\to {{\bf{Q}}}_{3}\cdots .$$

We solve the linear functions *r*_11_[1], *r*_22_[1], *s*_11_[1] and *s*_22_[1] in the scheme by directly solving the linear set of Eqs (17) and (18) with **Q**_0_, the first-order solutions for **Q**_1_ are given as following

22 $${{\rm{\Phi }}}_{+1}=[1+4\sqrt{2}\frac{{G}_{1}}{{D}_{1}}]\cdot {e}^{4i\tau (t)},$$

23 $${{\rm{\Phi }}}_{0}=[1+4\sqrt{2}\frac{{G}_{0}}{{D}_{1}}]\cdot {e}^{4i\tau (t)},$$

24 $${{\rm{\Phi }}}_{-1}=[-1+4\sqrt{2}\frac{{G}_{-1}}{{D}_{1}}]\cdot {e}^{4i\tau (t)},$$

with the functions *D*_1_, *G*_1_, *G*_0_ and *G*_−1_ in Eqs (22–24) are given in the Appendix B of Supplementary information. The solutions(22), (23) and (24) include the arbitrary real constants *c*_1_, *c*_2_, *c*_3_ and *c*_4_.

Specifically, the first-order solutions for **Q**_1_ with four arbitrary constants *c*_1_ = 1, *c*_2_ = 0, *c*_3_ = 1 and *c*_4_ = 0 are given as following

25 $${{\rm{\Phi }}}_{+1}^{^{\prime} }=[1+4\sqrt{2}\frac{{G}_{1}}{{D}_{1}}]\cdot {e}^{4i\tau (t)},$$

26 $${{\rm{\Phi }}}_{0}^{^{\prime} }=[1+4\sqrt{2}\frac{{G}_{0}}{{D}_{1}}]\cdot {e}^{4i\tau (t)},$$

27 $${{\rm{\Phi }}}_{-1}^{^{\prime} }=[-1+4\sqrt{2}\frac{{G}_{-1}}{{D}_{1}}]\cdot {e}^{4i\tau (t)},$$

with the functions *D*_1_, *G*_1_, *G*_0_ and *G*_−1_ in Eqs (25–27) are given in the Appendix C of Supplementary information.

**Figure 3:**
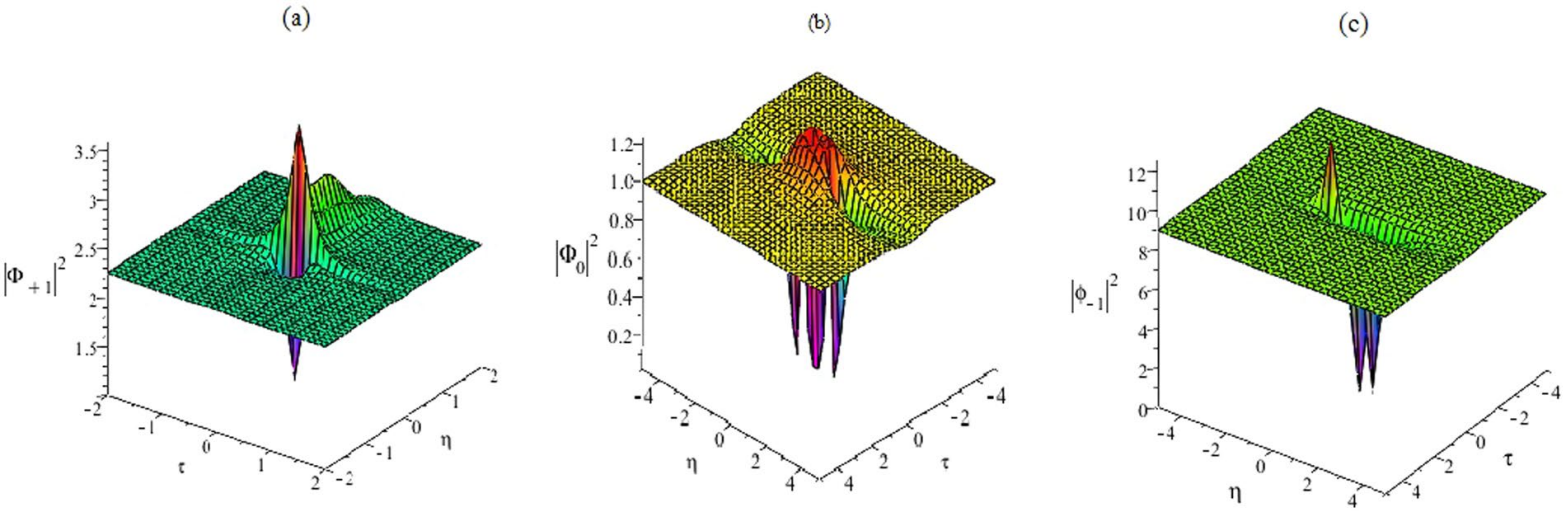
The first rational solution. (**a**) The first rational solution |Φ_+1_|^2^ of Eq. (25). (**b**) The first rational solution |Φ_0_|^2^ of Eq. (26). (**c**) The first rational solution |Φ_−1_|^2^ of Eq. (27).

It is easy to see that the rational-like solutions (25)–(27) are different from the known rational solutions of the CNLS Eq. 56, since it contains four parameters, which will generate abundant structures related to the optical rogue waves in Fig. 3.

**Figure 4:**
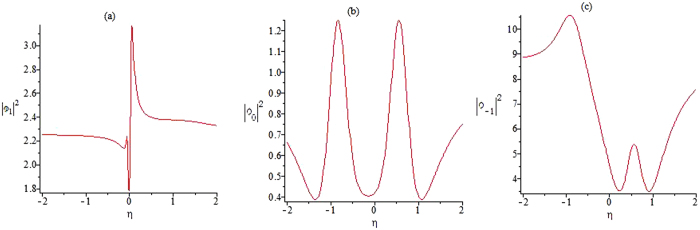
The amplitude of the first rogue wave solution. Plots of the amplitudes of the first rogue wave solutions |*ϕ*_1_|^2^, |*ϕ*_0_|^2^, |*ϕ*_−1_|^2^ in Eqs (25–27) on the central line *t* = 0.

Figure 4 gives the plots of the functions |*ϕ*_1_|^2^, |*ϕ*_0_|^2^ and |*ϕ*_−1_|^2^ on the central line t = 0, and it shows that the amplitudes are not symmetrical. The function |*ϕ*_1_|^2^ has two peaks. The higher peak has an approximate amplitude of 3.6179 and the lower one has an amplitude of 1.3672. The function |*ϕ*_0_|^2^ has four zeros and two peaks, whose peak amplitudes are close to the background amplitude. The function |*ϕ*_−1_|^2^ has different locations for peaks.

**Figure 5:**
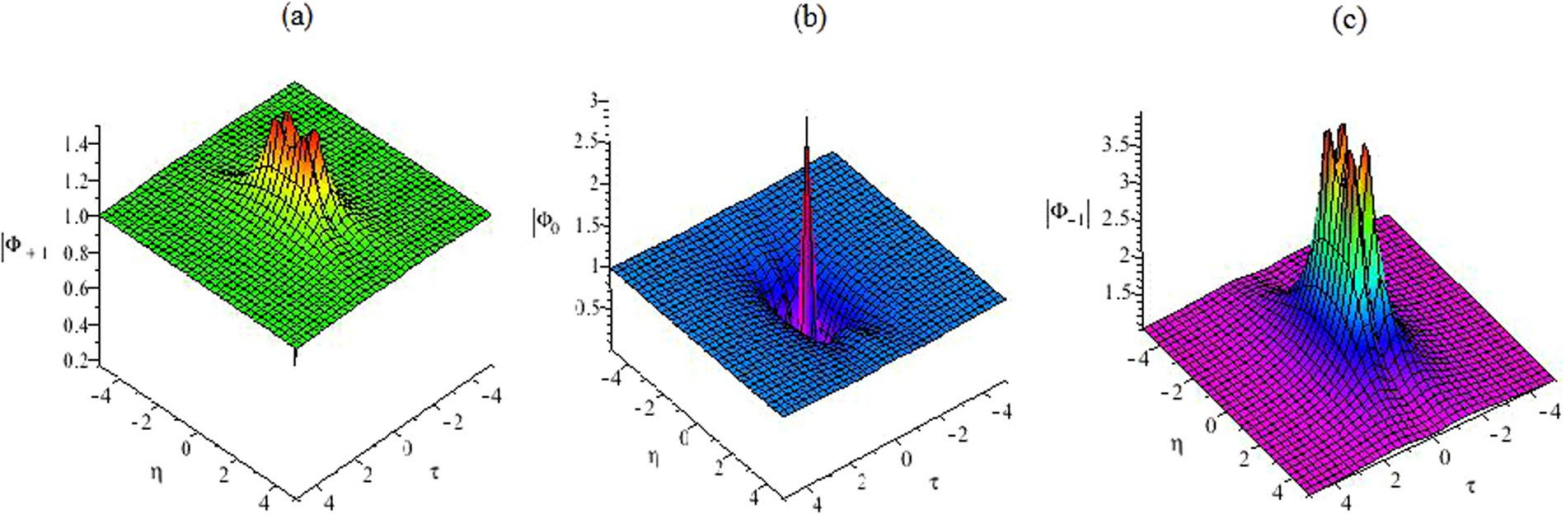
The second rational solution. (**a**) The second rational solution |Φ_+1_| of Eq. (28). (**b**) The second rational solution |Φ_0_| of Eq. (29). (**c**) The second rational solution |Φ_−1_| of Eq. (30).

We search for the functions *r*_11_[2], *r*_22_[2], *s*_11_[2] and *s*_22_[2] in this scheme by directly solving the linear set of Eqs (17) and (18) with **Q**_1_ function at the previous step, the second-order solutions for **Q**_2_ with arbitrary constants are given. Specifically, the second-order solutions for ${{\bf{Q}}}_{2}^{^{\prime} }$ are shown in Fig. 5 as following (*c*_1_ = 1, *c*_2_ = 1, *c*_3_ = 1, *c*_4_ = 1),

28 $${{\rm{\Phi }}}_{+1}=[-\,1-\frac{1}{4}\frac{{H}_{1}}{{D}_{2}}]\cdot {e}^{4i\tau (t)},$$

29 $${{\rm{\Phi }}}_{0}=[-\,1-\frac{1}{2}\frac{{H}_{0}}{{D}_{2}}]\cdot {e}^{4i\tau (t)},$$

30 $${{\rm{\Phi }}}_{-1}=[-1-\frac{1}{4}\frac{{H}_{-1}}{{D}_{2}}]\cdot {e}^{4i\tau (t)},$$

where

$$\begin{array}{rcl}{H}_{1} & = & (2-2\,\sqrt{2})\,(\mathrm{3/8}-6\,{\eta }^{2}-8\,{\eta }^{4}-144\,{\tau }^{2}-2560\,{\tau }^{4}-384\,{\eta }^{2}{\tau }^{2}\\  &  & +i(\frac{15}{4}+12\,{\eta }^{2}-16\,{\eta }^{4}-32\,{\tau }^{2}-1024\,{\tau }^{4}-256\,{\eta }^{2}{\tau }^{2})),\end{array}$$

$$\begin{array}{rcl}{H}_{0} & = & (\mathrm{3/8}-6\,{\eta }^{2}-8\,{\eta }^{4}-144\,{\tau }^{2}-2560\,{\tau }^{4}-384\,{\eta }^{2}{\tau }^{2}\\  &  & +i(\frac{15}{4}+12\,{\eta }^{2}-16\,{\eta }^{4}-32\,{\tau }^{2}-1024\,{\tau }^{4}-256\,{\eta }^{2}{\tau }^{2})),\end{array}$$

$$\begin{array}{rcl}{H}_{-1} & = & (-2-2\,\sqrt{2})\,(\mathrm{3/8}-6\,{\eta }^{2}-8\,{\eta }^{4}-144\,{\tau }^{2}-2560\,{\tau }^{4}-384\,{\eta }^{2}{\tau }^{2}\\  &  & +i(\frac{15}{4}+12\,{\eta }^{2}-16\,{\eta }^{4}-32\,{\tau }^{2}-1024\,{\tau }^{4}-256\,{\eta }^{2}{\tau }^{2})),\end{array}$$

$$\begin{array}{rcl}{D}_{2} & = & (\frac{3}{32}+\mathrm{9/4}\,{\eta }^{2}+2\,{\eta }^{4}+\mathrm{16/3}\,{\eta }^{6}+66\,{\tau }^{2}+1152\,{\tau }^{4}\\  &  & +\frac{8192}{3}\,{\tau }^{6}-96\,{\eta }^{2}{\tau }^{2}+128\,{\eta }^{4}{\tau }^{2}+1024\,{\eta }^{2}{\tau }^{4}).\end{array}$$

**Figure 6:**
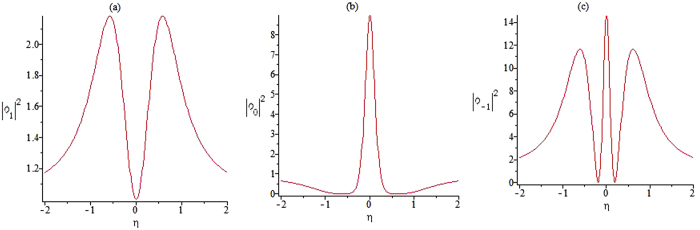
The amplitude of the second rogue wave solution. Plots of the amplitudes of the second rogue wave solutions |*ϕ*_1_|^2^, |*ϕ*_0_|^2^, |*ϕ*_−1_|^2^ in Eqs (28–30) on the central line *t* = 0.

It is clear that the rational-like solutions (28–30) are same to the rational solutions of the CNLS Eq. 56, the profile of the function *ϕ*_0_ has similar characteristics as the solution of the NLS equation except higher amplitude and location of zeros, while the profile of function *ϕ*_−1_ exhibits some interesting features different from the NLS equation. However, it is interesting that the profile of function *ϕ*_+1_ possesses a dark rogue wave role in Fig. 6.

### Non-autonomous multi-rogue wave solution and management for Eq. (1)

**Figure 7:**
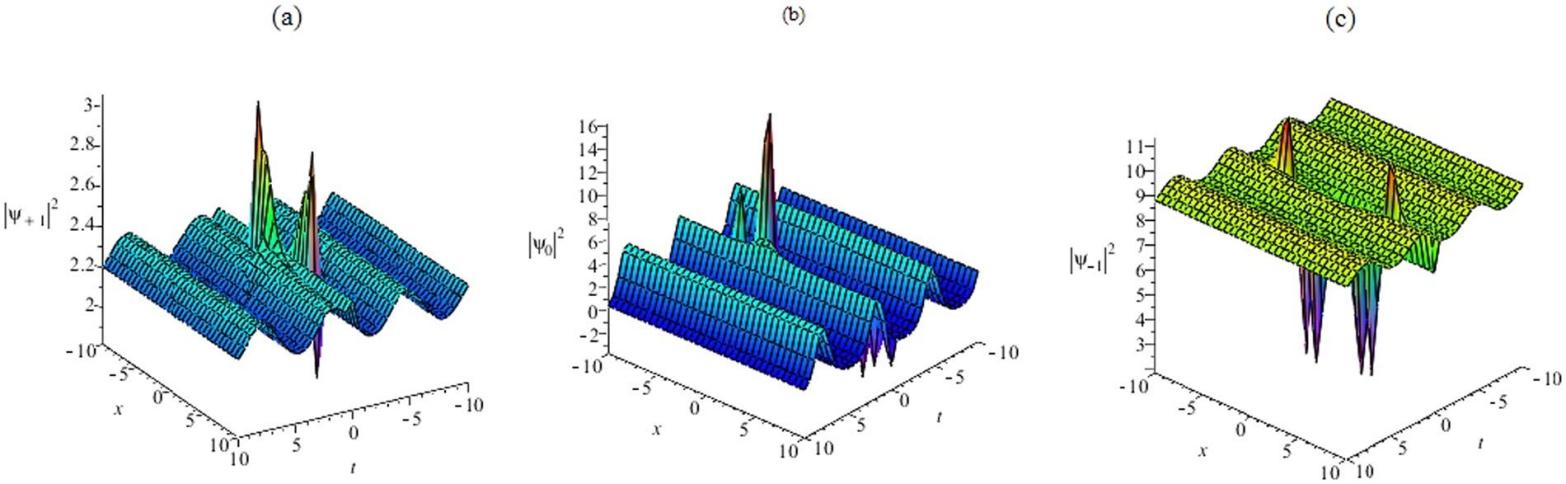
The first-order non-autonomous rogue wave. (**a**) The first-order non-autonomous rogue wave solution |Ψ_+1_|^2^ of Eq. (31) with Eq. (25), Γ(*t*) = 0.02cos*t*, *ρ*_0_ = 1, *α*(*t*) = sin(*t*), *b*(*t*) = cos(*t*), *a* = 1, *c* = 1. (**b**) The first-order non-autonomous rogue wave solution |Ψ_0_|^2^ of Eq. (31) with Eq. (26), Γ(*t*) = 0.8cost, *ρ*_0_ = 1, *α*(*t*) = sin(*t*), *b*(*t*) = cos(*t*), *a* = 1, *c* = 1. (**c**) The first-order non-autonomous rogue wave solution |Ψ_−1_|^2^ of Eq. (31) with Eq. (27), Γ(*t*) = 0.02cos*t*, *ρ*_0_ = 1, *α*(*t*) = sin(*t*), *b*(*t*) = cos(*t*), *a* = 1, *c* = 1.

In the following section, we also suggest a mechanism to tame the non-autonomous rogue waves by manipulating the external potential. We consider the management and dynamic of the non-autonomous rogue wave under time-dependent quadratic potential. As two representative examples, we use the first order and second order rational solutions of the CNLS equation. Based on the similarity transformation(3) and the rational solutions (25–27), we obtain the first-order non-autonomous rogue wave solutions of Eq. (1) in the forms

31 $${{\rm{\Psi }}}_{m}(x,t)\,\mathrm{[1]}={\rho }_{0}\,{{\rm{\Phi }}}_{m}\mathrm{[1]}\,(\eta (x,t),\tau (t)){e}^{{\int }_{0}^{t}{\rm{\Gamma }}(s)ds+i(-\tfrac{1}{2}{(\mathrm{ln}\alpha (t))}_{t}{x}^{2}-\tfrac{a{b}_{t}(t)}{\alpha (t)}x+d)},m=0,\pm 1,$$

with *η*(*x*, *t*) = *α*(*t*)*x* + *ab*(t) + *c* and $\tau (t)={\int }_{0}^{t}\,{\alpha }^{2}(s)ds$. We choose the free functions *α*(*t*), *b*(*t*), Γ(*t*) of time *t*, the Fig. 7 depicts the dynamical behaviors of the rogue wave solutions(31). Where *α*(*t*) can be associated with the inverse of the pulse width, *b*(*t*) represents the position of its center of mass, and c is a free constant.

To manipulate the rogue wave under the quadratic potential, we choose the arbitrary functions *α*(*t*) = sin(*t*), *b*(*t*) = cos(*t*), *a* = 1, *c* = 1 in Eq. (31) and derive the non-autonomous rogue wave solutions for the three-component GP equation in Fig. 7. According to Fig. 7(a–c), we find that the modulation of the amplitude function Γ(t) affects only the location of the peak. The peak of the pulse is obtained at *t* = 0. The higher peak has an approximate amplitude of 15.51 and the lower one has an amplitude of 1.26.

**Figure 8:**
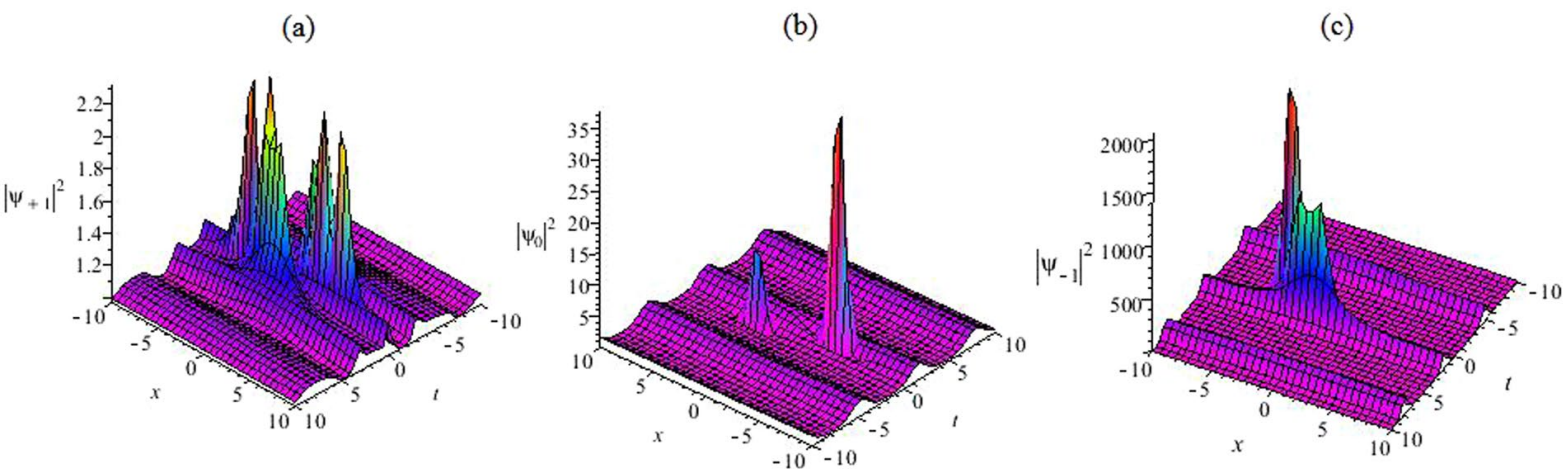
The second order non-autonomous rogue wave. (**a**) The second order non-autonomous rogue wave solution |Ψ_+1_|^2^ of Eq. (32) with Eq. (28), Γ(*t*) = 0.02cos*t*, *ρ*_0_ = 1, *α*(*t*) = sin(*t*), *b*(*t*) = cos(*t*), *a* = 1, *c* = 1. (**b**) The second order non-autonomous rogue wave |Ψ_0_|^2^ of Eq. (32) with Eq. (29), Γ(*t*) = 0.5cos*t*, *ρ*_0_ = 1, *α*(*t*) = sin(*t*), *b*(*t*) = cos(*t*), *a* = 1, *c* = 1. (**c**) The second order non-autonomous rogue wave solution |Ψ_−1_|^2^ of Eq. (32) with Eq. (30), Γ(*t*) = 2.5cos*t*, *ρ*_0_ = 1, *α*(*t*) = sin(*t*), *b*(*t*) = cos(*t*), *a* = 1, *c* = 1.

We use the second order rational solutions(28)–(30) of Eq. (2). As a result, we obtain the second-order non-autonomous rogue wave solutions of Eq. (1) in the forms

32 $${{\rm{\Psi }}}_{m}(x,t)\,\mathrm{[2]}={\rho }_{0}\,{{\rm{\Phi }}}_{m}\mathrm{[2]}\,(\eta (x,t),\tau (t)){e}^{{\int }_{0}^{t}{\rm{\Gamma }}(s)ds+i(-\frac{1}{2}{(\mathrm{ln}\alpha (t))}_{t}{x}^{2}-\frac{a{b}_{t}(t)}{\alpha (t)}x+d)},m=0,\pm 1,$$

with *η*(*x*, *t*) = *α*(*t*)*x* + *ab*(*t*) + *c* and $\tau (t)={\int }_{0}^{t}\,{\alpha }^{2}(s)ds$. If we choose different free functions *α*(*t*), *b*(*t*), Γ(*t*), the Fig. 8 depicts the dynamical behaviors of the multi-rogue wave solutions(32) with different parameters.

Figure 8 shows that their interactions and the peaks of the pulse at *t* = 0 in the three-component GP equation. The higher peak has an approximate amplitude of 2000, and the lower one has an amplitude of 2.26. In Fig. 8(a–c), the functions *η*(*x*, *t*) and *τ*(*t*) affect the wave shapes of peak. As a specific example, we consider the case of an oscillating harmonic potential of *α*(*t*). The peak intensity of the non-autonomous rogue wave soliton is located at *t* = 0. The background is obtained in the large t-limit. Noting that while *α*(*t*) is bounded, the integral *ρ*(*t*) is not bounded in the large t-limit, the background intensity will be given by ${\rho }_{0}{e}^{{\int }_{0}^{t}{\rm{\Gamma }}(s)ds}$. This may be useful to generate novel experimental results.

Based on Eqs (31) and (32), we find that the function *φ*(*x*, *t*) appears only in the phase factor, and it dose not effect on the property of the rogue wave and its location. We point out the importance of the two arbitrary functions *α*(*t*) and *b*(*t*) in Eqs (31) and (32), the *α*(*t*) can be associated with the inverse of the pulse width, the b(t) represents the position of its center of mass.

Figures 7 and 8 show such a non-autonomous multi-rogue wave solution as a result of an oscillating quadratic potential ${V}_{m}(x,t)=-\frac{1}{2}{(\mathrm{ln}\alpha (t))}_{tt}{x}^{2}$ − ${(\frac{a{b}_{t}(t)}{\alpha (t)})}_{t}x$ + $\frac{1}{2}{(-{(\mathrm{ln}\alpha (t))}_{t}x-\frac{a{b}_{t}(t)}{\alpha (t)})}^{2}$. Fast oscillations with $\rho (t)={\rho }_{0}{e}^{{\int }_{0}^{t}{\rm{\Gamma }}(s)ds}$ show that the non-autonomous rogue wave retrieves back its density profile for the inhomogeneous case, apart from some small amplitude fringes. They show such a focused multi-rogue wave soliton as a result of an oscillating quadratic potential. The non-autonomous multi-rogue wave solutions possess arbitrary functions, which are possible to control the rogue waves. The non-autonomous multi-rogue wave solutions (31) and (32) of Eq. (1) are localized both in time and in space and reveal the usual “rogue wave” features. We choose different free functions and parameters, the Figs 7 and 8 depict the different dynamical behaviors of the rogue waves.

These figures of the non-autonomous multi-rogue wave solutions (31) and (32) are different from the known rational solutions of the higher-order NLS equation^55, 56, 58^, and may be useful to raise the possibility of relative experiments and potential applications for the rogue wave phenomena. It is noted that Eq. (32) can also describe the dynamics of non-autonomous matter-wave solitons in BECs, where the soliton management can be realized by adjusting the related control parameters via the technique Feshbach resonance. This may provide the way to design external potential and to control soliton in nonlinear systems.

## Discussion

In this paper, we consider the effect of time-dependent quadratic potential on the structure and dynamic of rogue waves. We use the similarity reduction of the three-component coupled nonlinear GP equation to CNLS equation. We investigate some rogue wave solutions in a three component CNLS equation using the Darboux transformation method. We find the external potential of quadratic and some novel spatial temporal structures for non-autonomous multi-rogue wave solutions in the coupled system. The coupled system can be used to describe three-component BECs, multi-mode optical transmission, and so on. We obtain some novel spatial temporal structures for single, double and triple vector RWs in the coupled system, our method can be applied to the *F* = 2 spinor BECs. These obtained solutions can be used to describe the possible formation mechanisms for optical, oceanic and matter rogue wave phenomenon in optical fibre, the deep ocean and BECs, respectively.

## Methods

### New rational solutions by employing the Darboux transformation

It is well known that the Darboux transformation can apply directly to obtain the rogue wave solutions for the nonlinear evolution equations. We take the DT technique to construct the new rational solutions with arbitrary constants for the CNLS Eq. (2).

Now we recommend a gauge transformation **T** of the matrix NLS Eq. (9):

33 $$\begin{array}{l}\tilde{{\boldsymbol{\Psi }}}=T{\boldsymbol{\Psi }},{\bf{T}}=(\begin{array}{cc}{{\bf{T}}}_{11} & {{\bf{T}}}_{12}\\ {{\bf{T}}}_{21} & {{\bf{T}}}_{22}\end{array}),\end{array}$$

and

34 $$\begin{array}{l}{{\boldsymbol{\Psi }}}_{\eta }=\tilde{{\bf{U}}}\,{\boldsymbol{\Psi }},\,\tilde{{\bf{U}}}=({{\bf{T}}}_{\eta }+{\bf{TU}})\,{{\bf{T}}}^{-1},\end{array}$$

35 $${{\boldsymbol{\Psi }}}_{\tau }=\tilde{{\bf{V}}}\,{\boldsymbol{\Psi }},\,\tilde{{\bf{V}}}=({{\bf{T}}}_{\tau }+{\bf{TV}}){{\bf{T}}}^{-1}\mathrm{.}$$

If the $\tilde{{\bf{U}}}$, $\tilde{{\bf{V}}}$ and **U**, **V** have the same types, the system (33) is called Darboux transformation of the matrix NLS Eq. (9).

Let **Ψ** = (**Ψ**_1_, **Ψ**_2_)^*T*^, **Φ** = (**Φ**_1_, **Φ**_2_)^*T*^ are two basic solutions of the systems (34) and (35), then we give the following linear algebraic systems:

36 $$\begin{array}{l}\{\begin{array}{l}\sum _{i\mathrm{=0}}^{N-1}\,({{\bf{A}}}_{11}^{(i)}+{{\bf{A}}}_{12}^{(i)}{{\bf{M}}}_{j})=-{\lambda }_{j}^{N}I,\\ \sum _{i\mathrm{=0}}^{N-1}\,({{\bf{A}}}_{21}^{(i)}+{{\bf{A}}}_{22}^{(i)}{{\bf{M}}}_{j})=-{\lambda }_{j}^{N}{{\bf{M}}}_{j},\end{array}\end{array}$$

with

37 $${{\bf{M}}}_{j}=({{\boldsymbol{\Psi }}}_{2}+{\nu }_{j}\,{{\boldsymbol{\Phi }}}_{2})\,{({{\boldsymbol{\Psi }}}_{1}+{\nu }_{j}{{\boldsymbol{\Phi }}}_{1})}^{-1}\mathrm{,0}\le j\le 2N,$$

where *λ*_*j*_ and *ν*_*j*_ (*λ*_*i*_ ≠ *λ*_*j*_) should choose appropriate parameters, thus the determinants of coefficients for Eq. (45) are nonzero.

Defining a 4 × 4 matrix T, and T is of the form as following

38 $$\{\begin{array}{ll}{{\bf{T}}}_{11}={\lambda }^{N}I+\sum _{i\mathrm{=0}}^{N-1}\,{{\bf{A}}}_{11}^{(i)}{\lambda }^{i}, & {{\bf{T}}}_{12}=\sum _{i=0}^{N-1}\,{{\bf{A}}}_{12}^{(i)}{\lambda }^{i},\\ {{\bf{T}}}_{21}=\sum _{i\mathrm{=0}}^{N-1}\,{{\bf{A}}}_{21}^{(i)}{\lambda }^{i}, & {{\bf{T}}}_{22}={\lambda }^{N}I+\sum _{i=0}^{N-1}\,{{\bf{A}}}_{22}^{(i)}{\lambda }^{i},\end{array}$$

where *N* is a natural number, the *I* is an 2 × 2 unit matrix, the ${{\bf{A}}}_{mn}^{i}(m,n=1,2,i\ge 0)$ are the 2 × 2 matrix functions of *x* and *t*. Through calculations, we can obtain Δ**T** as following

39 $$\begin{array}{l}{\rm{\Delta }}{\bf{T}}=\prod _{j=1}^{2N}\,(\lambda -{\lambda }_{j}),\end{array}$$

which proves that *λ*_*j*_ (1 ≤ *j* ≤ 2*N*,) are 2*N* roots of Δ**T**. Based on these conditions, we will proof that the $\tilde{{\bf{U}}}$ and $\tilde{{\bf{V}}}$ have the same forms with **U** and **V**, respectively.

**Proposition 1**. The matrix $\tilde{{\bf{U}}}$ defined by (34) has the same type as **U**, that is,

40 $$\tilde{{\bf{U}}}=(\begin{array}{cc}\lambda I & \tilde{{\bf{Q}}}\\ -{\tilde{{\bf{Q}}}}^{\ast } & -\lambda I\end{array}),$$

in which the transformation formulas between old and new potentials are shown on as following

41 $$\begin{array}{l}\{\begin{array}{l}\,\,\tilde{{\bf{Q}}}={\bf{Q}}-2{{\bf{A}}}_{12},\\ {\tilde{{\bf{Q}}}}^{\ast }={{\bf{Q}}}^{\ast }-2{{\bf{A}}}_{21}.\end{array}\end{array}$$

The transformation (41) is used to get a DT of the spectral problem (34).

**Proof**. By assuming ${{\bf{T}}}^{-1}=\frac{{{\bf{T}}}^{\ast }}{{\rm{\Delta }}{\bf{T}}}$ and

42 $$({{\bf{T}}}_{x}+{\bf{TU}}){{\bf{T}}}^{\ast }=(\begin{array}{cc}{{\bf{B}}}_{11}(\lambda ) & {{\bf{B}}}_{12}(\lambda )\\ {{\bf{B}}}_{21}(\lambda ) & {{\bf{B}}}_{22}(\lambda )\end{array}),$$

it is easy to verify that **B**_*sl*_ (1 ≤ s, *l* ≤ 2) are 2N-order or 2N + 1-order polynomials in *λ*.

Through some accurate calculations, *λ*_*j*_ (1 ≤ *j* ≤ 2) are the roots of **B**_*sl*_ (1 ≤ *s*, *l* ≤ 2). Thus, Eq. (42) has the following structure

43 $$({{\bf{T}}}_{\eta }+{\bf{TU}}){{\bf{T}}}^{\ast }=({\rm{\Delta }}{\bf{T}}){\bf{C}}(\lambda )$$

where

44 $${\bf{C}}(\lambda )=(\begin{array}{cc}{{\bf{C}}}_{11}^{\mathrm{(1)}}\lambda +{{\bf{C}}}_{11}^{\mathrm{(0)}} & {{\bf{C}}}_{12}^{\mathrm{(0)}}\\ {{\bf{C}}}_{21}^{\mathrm{(0)}} & {{\bf{C}}}_{22}^{\mathrm{(1)}}\lambda +{{\bf{C}}}_{22}^{\mathrm{(0)}}\end{array}),$$

and ${{\bf{C}}}_{mn}^{(k)}$ (*m*, *n* = 1, 2, *k* = 0, 1) satisfy the functions without *λ*. The Eq. (43) is obtained as following

45 $$({{\bf{T}}}_{\eta }+{\bf{TU}})={\bf{C}}(\lambda ){\bf{T}}.$$

Through comparing the coefficients of *λ* in Eq. (45), we can obtain

46 $$\{\begin{array}{lll}{{\bf{C}}}_{11}^{\mathrm{(1)}}=I, & {{\bf{C}}}_{11}^{\mathrm{(0)}}={\bf{O}}, & {{\bf{C}}}_{12}^{\mathrm{(0)}}={\bf{Q}}-2{{\bf{A}}}_{12}=\tilde{{\bf{Q}}}\\ {{\bf{C}}}_{21}^{\mathrm{(0)}}=-{{\bf{Q}}}^{\ast }+2{{\bf{A}}}_{21}=-{\tilde{{\bf{Q}}}}^{\ast }, & {{\bf{C}}}_{22}^{\mathrm{(1)}}=-I, & {{\bf{C}}}_{22}^{\mathrm{(0)}}={\bf{O}}.\end{array}$$

In the following section, we assume that the new matrix $\tilde{{\bf{U}}}$ has the same type with **U**, which means that they have the same structures only **Q** and **Q** ^*^ of U transformed into $\tilde{{\bf{Q}}}$ and ${\tilde{{\bf{Q}}}}^{\ast }$ of $\tilde{{\bf{U}}}$. After careful calculation, we compare the ranks of *λ* ^*N*^, and get the objective equations as following:

47 $$\{\begin{array}{l}\tilde{{\bf{Q}}}={\bf{Q}}-2{{\bf{A}}}_{12},\\ {\tilde{{\bf{Q}}}}^{\ast }={{\bf{Q}}}^{\ast }-2{{\bf{A}}}_{21},\end{array}$$

from Eqs (34) and (45), we know that $\tilde{{\bf{U}}}={\bf{C}}(\lambda )$. The proof is completed.

**Proposition 2**. Under the transformation (41), the matrix $\tilde{{\bf{V}}}$ defined by (35) has the same form as **V**, that is,

48 $$\begin{array}{l}\tilde{{\bf{V}}}=(\begin{array}{ll}2i{\lambda }^{2}I+i\tilde{{\bf{Q}}}{\tilde{{\bf{Q}}}}^{\ast } & 2\tilde{{\bf{Q}}}\lambda +i{\tilde{{\bf{Q}}}}_{\eta }\\ -2i\lambda {\tilde{{\bf{Q}}}}^{\ast }+i{\tilde{{\bf{Q}}}}_{\eta }^{\ast } & -2i{\lambda }^{2}I-i{\tilde{{\bf{Q}}}}^{\ast }\tilde{{\bf{Q}}}\end{array})\mathrm{.}\end{array}$$

**Proof**. We assume the new matrix $\tilde{{\bf{V}}}$ also has the same form with **V**. If we obtain the similar relations between **Q**, **Q** ^*^ and $\tilde{{\bf{Q}}},{\tilde{{\bf{Q}}}}^{\ast }$ in Eq. (41), we can prove that the gauge transformations under **T** turn the Lax pairs **U**, **V** into new Lax pairs $\tilde{{\bf{U}}},\tilde{{\bf{V}}}$ with the same types.

By assuming **T** ^−1^ = $\frac{{{\bf{T}}}^{\ast }}{{\rm{\Delta }}{\bf{T}}}$ and

49 $$({{\bf{T}}}_{\tau }+{\bf{TV}}){{\bf{T}}}^{\ast }=(\begin{array}{cc}{{\bf{E}}}_{11}(\lambda ) & {{\bf{E}}}_{12}(\lambda )\\ {{\bf{E}}}_{21}(\lambda ) & {{\bf{E}}}_{22}(\lambda )\end{array}),$$

it is easy to verify that **E**_*sl*_ (1 ≤ *s*, *l* ≤ 2) are 2N + 1-order or 2N + 2-order polynomials in *λ*.

Through some calculations, *λ*_j_ (1 ≤ *j* ≤ 2) are the roots of **E**_*sl*_ (1 ≤ *s*, *l* ≤ 2). Thus, Eq. (49) has the following structure

50 $$({{\bf{T}}}_{\tau }+{\bf{TV}}){{\bf{T}}}^{\ast }=({\rm{\Delta }}{\bf{T}})\,{\bf{F}}(\lambda ),$$

where

51 $${\bf{F}}(\lambda )=(\begin{array}{cc}{{\bf{F}}}_{11}^{\mathrm{(2)}}{\lambda }^{2}+{{\bf{F}}}_{11}^{\mathrm{(1)}}\lambda +{{\bf{F}}}_{11}^{\mathrm{(0)}} & {{\bf{F}}}_{12}^{\mathrm{(1)}}\lambda +{{\bf{F}}}_{12}^{\mathrm{(0)}}\\ {{\bf{F}}}_{21}^{\mathrm{(1)}}\lambda +{{\bf{F}}}_{21}^{\mathrm{(0)}} & {{\bf{F}}}_{22}^{\mathrm{(2)}}{\lambda }^{2}+{{\bf{F}}}_{22}^{\mathrm{(1)}}\lambda +{{\bf{F}}}_{22}^{\mathrm{(0)}}\end{array}),$$

and ${{\bf{F}}}_{mn}^{(k)}(m,n=1,2,k=0,\mathrm{1)}$ satisfy the functions without *λ*. According to Eq. (50), the following equation is obtained

52 $$({{\bf{T}}}_{\tau }+{\bf{TV}})={\bf{F}}(\lambda ){\bf{T}}.$$

Through comparing the coefficients of *λ* in Eq. (52), we get the objective equations as following:

53 $$\{\begin{array}{l}{{\bf{F}}}_{11}^{(2)}=2iI,{{\bf{F}}}_{11}^{(1)}={\bf{O}},{{\bf{F}}}_{11}^{(0)}=i{\bf{Q}}{{\bf{Q}}}^{\ast }{\boldsymbol{+}}2i{{\bf{Q}}}^{\ast }\,{{\bf{A}}}_{12}-2i\tilde{{\bf{Q}}}\,{{\bf{A}}}_{21},\\ {{\bf{F}}}_{12}^{(1)}=2i{\bf{Q}}-4i{{\bf{A}}}_{12}=2i\tilde{{\bf{Q}}},{{\bf{F}}}_{12}^{(0)}=i{{\bf{Q}}}_{x}+2i{{\bf{A}}}_{11}{\bf{Q}}-2i\tilde{{\bf{Q}}}\,{{\bf{A}}}_{22},\\ {{\bf{F}}}_{21}^{(1)}=-2i\tilde{{\bf{Q}}}+4{{\bf{A}}}_{21}=-2i{\tilde{{\bf{Q}}}}^{\ast },{{\bf{F}}}_{21}^{(0)}=i\tilde{{{\bf{Q}}}_{x}^{\ast }}-2i{{\bf{Q}}}^{\ast }\,{{\bf{A}}}_{22}+2i{\tilde{{\bf{Q}}}}^{\ast }\,{{\bf{A}}}_{11},\\ {{\bf{F}}}_{22}^{(2)}=-2iI,{{\bf{F}}}_{22}^{(1)}={\bf{O}},{{\bf{F}}}_{22}^{(0)}=-i{{\bf{Q}}}^{\ast }\,{\bf{Q}}+2i{\bf{Q}}{{\bf{A}}}_{21}+2i{\tilde{{\bf{Q}}}}^{\ast }.\end{array}$$

In the above section, we assume the new matrix $\tilde{{\bf{V}}}$ has the same type with **V**, which means they have the same structures only **Q** and **Q** ^*^ of **V** transformed into $\tilde{{\bf{Q}}}$ and ${\tilde{{\bf{Q}}}}^{\ast }$ of $\tilde{{\bf{V}}}$. From Eqs (41) and (53), we know that $\tilde{{\bf{V}}}={\bf{F}}(\lambda )$. The proof is completed.

The Propositions 1 and 2 show that the transformations (33) and (41) are Darboux transformations connecting matrix NLS equation. In what follows, we can apply the above DT (41) to construct exact solutions of matrix NLS equation. Firstly, we give a set of seed solutions and substitute the solutions into Eqs (34) and (35), we will get the basic solutions for these equations:

54 $${\rm{\Psi }}(\lambda )=(\begin{array}{c}{\psi }_{2}\\ {\psi }_{2}\end{array}),\,{\rm{\Phi }}(\lambda )=(\begin{array}{c}{\varphi }_{1}\\ {\varphi }_{2}\end{array}).$$

In order to calculate, we consider *N* = 1 in Eqs (36) and (38), and obtain the matrix T

55 $$\begin{array}{l}{\bf{T}}=(\begin{array}{cc}\lambda I+{{\bf{A}}}_{11} & {{\bf{A}}}_{12}\\ {{\bf{A}}}_{21} & \lambda I+{{\bf{A}}}_{22}\end{array}),\end{array}$$

and

56 $$\{\begin{array}{l}{\lambda }_{j}I+{{\bf{A}}}_{11}+{{\bf{M}}}_{j}{{\bf{A}}}_{12}={\bf{O}},\\ {{\bf{A}}}_{21}{\boldsymbol{+}}{{\bf{M}}}_{j}({{\boldsymbol{\lambda }}}_{j}+{{\bf{A}}}_{22})={\bf{O}}.\end{array}$$

According to Eq. (56), we get

57 $$\begin{array}{l}{\rm{\Delta }}[1]=|\begin{array}{cc}I & {M}_{1}\\ I & {M}_{2}\end{array}|,\,{{\rm{\Delta }}}_{12}[1]=|\begin{array}{cc}I & -{\lambda }_{1}I\\ I & -{\lambda }_{2}I\end{array}|,\end{array}$$

based on Eqs (56) and (57), we can obtain the following system

58 $${{\bf{A}}}_{12}=\frac{{{\rm{\Delta }}}_{12}[1]}{{\rm{\Delta }}[1]},$$

the analytic soliton solutions of matrix NLS equation are obtained by the DT method(41) as following

59 $${\tilde{{\bf{Q}}}}_{1}={{\bf{Q}}}_{0}-2{{\bf{A}}}_{12}.$$

In order to obtain the *n*–*th* iteration of the DT, we consider *N* = *n* in Eqs (36) and (38), and obtain the matrix **T**

60 $${\bf{T}}=(\begin{array}{cc}{\lambda }^{n}I+{{\rm{\Sigma }}}_{i=0}^{N-1}\,{{\bf{A}}}_{11}^{i}{\lambda }_{j}^{i} & {{\rm{\Sigma }}}_{i=0}^{N-1}\,{{\bf{A}}}_{12}^{i}{\lambda }_{j}^{i}\\ {{\rm{\Sigma }}}_{i=0}^{N-1}\,{{\bf{A}}}_{21}^{i}{\lambda }_{j}^{i} & {\lambda }^{n}I+{{\rm{\Sigma }}}_{i=0}^{N-1}\,{{\bf{A}}}_{22}^{i}{\lambda }_{j}^{i}\end{array}),$$

and

61 $$\{\begin{array}{l}{\lambda }_{j}^{N}I+{{\rm{\Sigma }}}_{i\mathrm{=0}}^{N-1}({{\bf{A}}}_{11}^{i}+{{\bf{M}}}_{j}{{\bf{A}}}_{12}^{i}){\lambda }_{j}^{i}={\bf{O}},\\ {{\bf{M}}}_{j}{\lambda }_{j}^{N}+{{\rm{\Sigma }}}_{i=0}^{N-1}\,({{\bf{A}}}_{21}^{i}+{{\bf{M}}}_{j}{{\bf{A}}}_{22}^{i}){\lambda }_{j}^{i}={\bf{O}}.\end{array}$$

According to Eq. (61), we get

62 $${\rm{\Delta }}[N]=|\begin{array}{ccccccccc}I & {{\bf{M}}}_{1} & {\lambda }_{1}I & {{\bf{M}}}_{1}{\lambda }_{1} & {\lambda }_{1}^{2}I & {{\bf{M}}}_{1}{\lambda }_{1}^{2} & \cdots  & {\lambda }_{1}^{n-1}I & {{\bf{M}}}_{1}{\lambda }_{1}^{n-1}\\ I & {{\bf{M}}}_{2} & {\lambda }_{2}I & {{\bf{M}}}_{2}{\lambda }_{2} & {\lambda }_{2}^{2}I & {{\bf{M}}}_{2}{\lambda }_{2}^{2} & \cdots  & {\lambda }_{2}^{n-1}I & {{\bf{M}}}_{2}{\lambda }_{2}^{n-1}\\ \vdots  & \vdots  & \vdots  & \vdots  & \vdots  & \vdots  & \cdots  & \vdots  & \vdots \\ I & {{\bf{M}}}_{2n} & {\lambda }_{2n}I & {{\bf{M}}}_{2n}{\lambda }_{2n} & {\lambda }_{2n}^{2}I & {{\bf{M}}}_{2n}{\lambda }_{2n}^{2} & \cdots  & {\lambda }_{2n}^{n-1}I & {{\bf{M}}}_{2n}{\lambda }_{2n}^{n-1}\end{array}|,$$

$${{\rm{\Delta }}}_{12}[N]=|\begin{array}{ccccccccc}I & -{\lambda }_{1}^{n}I & {\lambda }_{1}I & {{\bf{M}}}_{1}{\lambda }_{1} & {\lambda }_{1}^{2}I & {{\bf{M}}}_{1}{\lambda }_{1}^{2} & \cdots  & {\lambda }_{1}^{n-1}I & {{\bf{M}}}_{1}{\lambda }_{1}^{n-1}\\ I & -{\lambda }_{2}^{n}I & {\lambda }_{2}I & {{\bf{M}}}_{2}{\lambda }_{2} & {\lambda }_{2}^{2}I & {{\bf{M}}}_{2}{\lambda }_{2}^{2} & \cdots  & {\lambda }_{2}^{n-1}I & {{\bf{M}}}_{2}{\lambda }_{2}^{n-1}\\ \vdots  & \vdots  & \vdots  & \vdots  & \vdots  & \vdots  & \cdots  & \vdots  & \vdots \\ I & -{\lambda }_{2n}^{n}I & {\lambda }_{2n}I & {{\bf{M}}}_{2n}{\lambda }_{2n} & {\lambda }_{2n}^{2}I & {{\bf{M}}}_{2n}{\lambda }_{2n}^{2} & \cdots  & {\lambda }_{2n}^{n-1}I & {{\bf{M}}}_{2n}{\lambda }_{2n}^{n-1}\end{array}|.$$

Based on Eqs (61) and (62), we can obtain the following system

63 $${{\bf{A}}}_{12}[N]=\frac{{{\rm{\Delta }}}_{12}[N]}{{\rm{\Delta }}[N]},$$

the analytic soliton solutions of matrix NLS Eq. (9) are obtained by *n*-*th* iteration of the DT as following

64 $${\tilde{{\bf{Q}}}}_{n}={{\bf{Q}}}_{0}-2{{\bf{A}}}_{12}[N].$$

We apply a simplify expression (16) from the *n*–*th* iteration of the DT (64) and choose a “seed solution” $${{\bf{Q}}}_{0}=(\begin{array}{cc}1 & 1\\ 1 & -1\end{array}){e}^{4i\tau }$$, the higher order rational solutions for the 3-component CNLS equation are derived in Eqs (22–30).

## Electronic supplementary material

Supplementary information

## References

[1] Akhmediev N, Ankiewicz A, Soto-Crespo JM (2009). Rogue waves and rational solutions of the nonlinear Schr*ö*dinger equation. Phys. Rev. E..

[2] Akhmediev N, Ankiewicz A, Taki M (2009). Waves that appear from nowhere and disappear without a trace. Phys. Lett. A..

[3] Ohta Y, Yang JK (2012). General high-order rogue waves and their dynamics in the nonlinear Schr*ö*dinger equation. Proce. Royal Soc. A..

[4] Ohta Y, Yang JK (2012). Dynamics of rogue waves in the Davey-Stewartson I equation. Phys. Rev. E..

[5] Ankiewicz, A. Rogue Ocean Waves. http://demonstrations.wolfram.com/Rogue Ocean Waves/2017/06/06/ URL of website (2009).

[6] Kibler B (2010). The Peregrine soliton in nonlinear fibre optics. Nat. Phys..

[7] Chabchoub A, Hoffmann NP, Akhmediev N (2011). Rogue Wave Observation in a Water Wave Tank. Phys. Rev. Lett..

[8] Ankiewicz A, Soto-Crespo JM, Akhmediev N (2010). Rogue waves and rational solutions of the Hirota equation. Phys. Rev. E..

[9] Voronovich VV, Shrira VI, Thomas G (2008). Can bottom friction suppress ‘freak wave’ formation. J. Fluid Mech..

[10] Stamper-Kurn DM (1998). Optical Confinement of a Bose-Einstein Condensate. Phys. Rev. Lett..

[11] Bloch I, Dalibard J, Zwerger W (2008). Many-body physics with ultracold gases. Rev. Mod. Phys..

[12] Solli DR, Ropers C, Koonath P, Jalali B (2007). Optical rogue waves. Nature..

[13] Fedichev PO, Kagan Y, Shlyapnikov GV, Walraven JT (1996). Influence of Nearly Resonant Light on the Scattering Length in Low-Temperature Atomic Gases. Phys. Rev. Lett..

[14] Theis M (2004). Tuning the scattering length with an optically induced Feshbach resonance. Phys. Rev. Lett..

[15] Papoular DJ, Shlyapnikov GV, Dalibard J (2010). Microwave-induced Fano-Feshbach resonances. Phys. Rev. A..

[16] Ho TL (1998). Spinor Bose Condensates in Optical Traps. Phys. Rev. Lett..

[17] Ohmi T, Machida K (1998). Bose-Einstein condensation with internal degrees of freedom in alkali atom gases. J. Phys. Soc. Jpn..

[18] Zhang W, Yi S, You L (2003). Mean field ground state of a spin-1 condensate in a magnetic field. New J. Phys..

[19] Barrett MD, Sauer JA, Chapman MS (2001). All-Optical Formation of an Atomic Bose-Einstein Condensate. Phys. Rev. Lett..

[20] Gorlitz A (2003). Sodium Bose-Einstein Condensates in the F = 2 State in a Large-Volume Optical Trap. Phys. Rev. Lett..

[21] Leanhardt AE (2003). Coreless Vortex Formation in a Spinor Bose-Einstein Condensate. Phys. Rev. Lett..

[22] Stenger J (1998). Spin domains in ground-state Bose-Einstein condensates. Nature..

[23] Miesner HJ (1999). Observation of Metastable States in Spinor Bose-Einstein Condensates. Phys. Rev. Lett..

[24] Kobayashi M, Kawaguchi Y, Nitta M, Ueda M (2009). Collision Dynamics and Rung Formation of non-Abelian Vortices. Phys. Rev. Lett..

[25] Baronio F, Degasperis A, Conforti M, Wabnitz S (2012). Solutions of the Vector Nonlinear Schrödinger Equations: Evidence for Deterministic Rogue Waves. Phys. Rev. Lett..

[26] Guo BL, Ling LMR (2011). Wave, Breathers and Bright-Dark-Rogue Solutions for the Coupled Schr*ö*dinger Equations. Chin. Phys. Lett..

[27] Bludov YV, Konotop VV, Akhmediev N (2010). Vector rogue waves in binary mixtures of Bose-Einstein condensates. Eur. Phys. J. Spec. Top..

[28] Zhao LC, Liu J (2012). Localized nonlinear waves in a two-mode nonlinear fiber. J. Opt. Soc. Am. B..

[29] Akhmediev, N. & Ankiewicz, A. Solitons: Nonlinear Pulses and Beams (Chapman and Hall Press, London, 1997).

[30] Kivshar, Y. S. & Agrawal, G. P. Optical Solitons: From Fibers to Photonic Crystals (Academic Press, New York, 2003).

[31] Barnett MP, Capitani JF, Von Zur Gathen J, Gerhard J (2004). Symbolic calculation in chemistry: Selected examples. Int. J. Quantum Chem..

[32] Matveev, V. B. & Salle, M. A. Darboux Transformation and Solitons (Springer Press, Berlin, 1991).

[33] Ablowitz, M. J. & Clarkson, P. A. Solitons, Nonlinear Evolution Equations and Inverse Scattering (Cambridge Univ Press, New York, 1991).

[34] Wadati M (1975). Wave propagation in nonlinear lattice. J. Phys. Soc. Jpn..

[35] Gao YT, Tian B (2007). Reply to: “Comment on: ‘Spherical Kadomtsev-Petviashvili equation and nebulons for dust ion-acoustic waves with symbolic computation”. Phys. Lett. A..

[36] Weiss J, Tabor M, Carnevale G (1983). The Painleve property for partial differential equations. J. Math. Phys..

[37] Hirota, R. The Direct Method in Soliton Theory (Cambridge Univ. Press, Cambridge, 2004).

[38] Carretero-Gonzalez R, Frantzeskakis DJ, Kevrekidis PG (2008). Nonlinear waves in Bose-Einstein condensates. Nonlinearity..

[39] Ma WX (1997). Darboux Transformations for a Lax Integrable System in 2n Dimensions. Lett. Math. Phys..

[40] Ma WX, You YC (2004). Rational solutions of the Toda lattice equation in Casoratian form. Chaos Solitons Fract..

[41] Ma WX, Chen M (2009). Direct search for exact solutions to the nonlinear Schrödinger equation. Appl. Math. Compu..

[42] Ma WX, Zhou Y, Dougherty R (2016). Lump-type solutions to nonlinear differential equations derived from generalized bilinear equations. Int. J. Mod. Phys. B..

[43] Matveev, V. B. & Salle, M. A. Darboux transformations and solitons (Springer-Verlag Press, Berlin, 1991).

[44] Matveev VB, Salle MA (1979). Differential-difference evolution equations. II (Darboux transformation for the Toda lattice). Lett. Math. Phys..

[45] Matveev VB, Salle MA, Rybin AV (1988). Darboux transformations and coherent interaction of the light pulse with two-level media. Inverse Problems..

[46] Babich VM, Matveev VB, Sail MA (1986). A binary Darboux transformation for the Toda lattice. J. Soviet. Math..

[47] Zakharov VE, Shabat AB (1974). A Scheme for Integrating Nonlinear Equations of Mathematical Physics by the Method of the Inverse Scattering Transform.I. Funct. Annal. and Appl..

[48] Zakharov VE, Shabat AB (1979). Integration of nonlinear equations of mathematical physics by the method of inverse scattering. II. Funct. Annal. and Appl..

[49] Mikhailov AV (1981). The reduction problem and the inverse scattering method. Phys. D..

[50] Mikhailov AV, Papamikos G, Wang JP (2016). Dressing method for the vector sine-Gordon equation and its soliton interactions. Phys. D..

[51] Bury R, Mikhailov AV, Wang JP (2017). Wave fronts and cascades of soliton interactions in the periodic two dimensional Volterra system. Phys. D..

[52] Mikhailov AV, Papamikos G, Wang JP (2016). Darboux transformation for the vector sine-Gordon equation and integrable equations on a sphere. Lett. Math. Phys..

[53] Yan ZY, Konotop VV (2009). Exact solutions to three-dimensional generalized nonlinear Schr*ö*dinger equations with varying potential and nonlinearities. Phys. Rev. E..

[54] Yu FJ (2016). Nonautonomous rogue waves and ‘catch’ dynamics for the combined Hirota-LPD equation with variable coefficients. Commun. Nonlinear. Sci. Numer. Simulat..

[55] Yu FJ (2013). Multi-rogue waves for a higher-order nonlinear Schrödinger equation in optical fibers. Appl. Math. Compu..

[56] Qin ZY, Mu G (2012). Matter rogue waves in an F = 1 spinor Bose-Einstein condensate. Phys. Rev. E..

[57] Li L (2005). Exact soliton solutions and nonlinear modulation instability in spinor Bose-Einstein condensates. Phys. Rev. A..

[58] Vijayajayanthi M, Kanna T, Lakshmanan M (2008). Bright-dark solitons and their collisions in mixed N-coupled nonlinear Schr*ö*dinger equations. Phys. Rev. A..

[59] Dai CQ, Zhou GQ, Zhang JF (2012). Controllable optical rogue waves in the femtosecond regime. Phys. Rev. E..

[60] He JS, Li YS (2011). Designable Integrability of the Variable-Coefficient Nonlinear Schr*ö*dinger Equations. Stud. Appl. Math..

[61] Ma WX, Zhou RG (2002). Adjoint symmetry constraints of multicomponent AKNS equations. Chinese Ann. Math. B..

