# Supplementary material for: Non-autonomous multi-rogue waves for spin-1 coupled nonlinear Gross-Pitaevskii equation and management by external potentials
Source: Sci Rep. 2017 Sep 6;7:10638. doi: 10.1038/s41598-017-10205-4 (PMC5587746; doi:10.1038/s41598-017-10205-4)
Supplement: Supplementary file 1 — Supplementary information [file 41598_2017_10205_MOESM1_ESM.pdf]

Supplementary information: Non-autonomous multi-rogue waves for spin-1 coupled nonlinear Gross-Pitaevskii equation and management by external potentials

Li Li   Fajun Yu *

School of Mathematics and Systematic Sciences, Shenyang Normal University, Shenyang 110034, China

Supplementary information

APPENDIX A: The 16 linear equations in Eqs.(17) and (18)

$r_{11,\eta}-\lambda\, r_{11}-\phi_1 s_{11}-\phi_0 s_{21}=0$, $r_{12,\eta}-\lambda\, r_{12}-\phi_1 s_{12}-\phi_0 s_{22}=0$, $r_{21,\eta}-\lambda\, r_{21}-\phi_0 s_{11}-\phi_{-1} s_{21}=0$, $r_{22,\eta}-\lambda\, r_{22}-\phi_0 s_{12}-\phi_{-1} s_{22}=0$, $s_{11,\eta}+\phi_{11} r_{11}+\phi_{10} r_{21}+\lambda\, s_{11}=0$, $s_{12,\eta}+\phi_{11} r_{12}+\phi_{10} r_{22}+\lambda\, s_{12}=0$, $s_{21,\eta}+\phi_{10} r_{11}+\phi_{-11} r_{21}+\lambda\, s_{21}=0$, $s_{22,\eta}+\phi_{10} r_{12}+\phi_{-11} r_{22}+\lambda\, s_{22}=0$,
and
$r_{11,\tau}-(2\,i\lambda^2+i\phi_1\phi_{11}+i\phi_0\phi_{10})r_{11}-i(\phi_1\phi_{10}+\phi_0\phi_{-11})r_{21}-i(2\,\lambda\,\phi_1+\phi_{1,x})s_{11}-i(2\,\lambda\,\phi_0+\phi_{0,x})s_{21}=0$,
$r_{12,\tau}-(2\,i\lambda^2+i\phi_1\phi_{11}+i\phi_0\phi_{10})r_{12}-i(\phi_1\phi_{10}+\phi_0\phi_{-11})r_{22}-i(2\,\lambda\,\phi_1+\phi_{1,x})s_{12}-i(2\,\lambda\,\phi_0+\phi_{0,x})s_{22}=0$,
$r_{21,\tau}-i(\phi_0\phi_{11}+\phi_{-1}\phi_{10})r_{11}-(2\,i\lambda^2+i\phi_0\phi_{10}+i\phi_{-1}\phi_{-11})r_{21}-i(2\,\lambda\,\phi_0+\phi_{0,x})s_{11}-i(2\,\lambda\,\phi_{-1}+\phi_{-1,x})s_{21}=0$,
$r_{22,\tau}-i(\phi_0\phi_{11}+\phi_{-1}\phi_{10})r_{12}-(2\,i\lambda^2+i\phi_0\phi_{10}+i\phi_{-1}\phi_{-11})r_{22}-i(2\,\lambda\,\phi_0+\phi_{0,x})s_{12}-i(2\,\lambda\,\phi_{-1}+\phi_{-1,x})s_{22}=0$,
$s_{11,\tau}-i(-2\,\lambda\,\phi_{11}+\phi_{11,x})r_{11}-i(-2\,\lambda\,\phi_{10}+\phi_{10,x})r_{21}-(-2\,i\lambda^2-i\phi_1\phi_{11}-i\phi_0\phi_{10})s_{11}+i(\phi_0\phi_{11}+\phi_{-1}\phi_{10})s_{21}=0$,
$s_{12,\tau}-i(-2\,\lambda\,\phi_{11}+\phi_{11,x})r_{12}-i(-2\,\lambda\,\phi_{10}+\phi_{10,x})r_{22}-(-2\,i\lambda^2-i\phi_1\phi_{11}-i\phi_0\phi_{10})s_{12}+i(\phi_0\phi_{11}+\phi_{-1}\phi_{10})s_{22}=0$,
$s_{21,\tau}-i(-2\,\lambda\,\phi_{10}+\phi_{10,x})r_{11}-i(-2\,\lambda\,\phi_{-11}+\phi_{-11,x})r_{21}+i(\phi_1\phi_{10}+\phi_0\phi_{-11})s_{11}-(-2\,i\lambda^2-i\phi_0\phi_{10}-i\phi_{-1}\phi_{-11})s_{21}=0$, $s_{22,\tau}-i(-2\,\lambda\,\phi_{10}+\phi_{10,x})r_{12}-i(-2\,\lambda\,\phi_{-11}+\phi_{-11,x})r_{22}+i(\phi_1\phi_{10}+\phi_0\phi_{-11})s_{12}-(-2\,i\lambda^2-i\phi_0\phi_{10}-i\phi_{-1}\phi_{-11})s_{22}=0$.

APPENDIX B: The functions D_1, G_1, G_0 and G_{-1} in Eqs.(22)-(24)

$D_1=\overline{s_{11}}\,\overline{s_{22}}s_{11}s_{22}-\overline{s_{11}}\,\overline{s_{22}}{s_{12}}^2+\overline{s_{11}}\,\overline{r_{21}}r_{21}s_{22}-\overline{s_{11}}\,\overline{r_{21}}r_{22}s_{12}-\overline{s_{11}}\,\overline{r_{22}}r_{21}s_{12}+\overline{s_{11}}\,\overline{r_{22}}r_{22}s_{11}+\overline{s_{22}}\,\overline{r_{12}}r_{12}s_{11}+\overline{s_{22}}\,\overline{r_{11}}r_{11}s_{22}-\overline{s_{22}}\,\overline{r_{11}}r_{12}s_{12}-\overline{s_{22}}\,\overline{r_{12}}r_{11}s_{12}-\overline{r_{12}}\,\overline{r_{21}}r_{11}r_{22}+\overline{r_{12}}\,\overline{r_{21}}r_{12}r_{21}+\overline{r_{22}}\,\overline{r_{11}}r_{11}r_{22}-$

*E-mail address: yufajun888@163.com

$\overline{r_{22}r_{11}}r_{12}r_{21}-\left(\overline{s_{12}}\right)^2 s_{11}s_{22}+\overline{s_{12}r_{12}}r_{21}s_{12}-\overline{s_{12}r_{12}}r_{22}s_{11}+\overline{s_{12}r_{22}}r_{11}s_{12}-\overline{s_{12}r_{22}}r_{12}s_{11}-\overline{s_{12}r_{11}}r_{21}s_{22}+\overline{s_{12}r_{11}}r_{22}s_{12}-\overline{s_{12}r_{21}}r_{11}s_{22}+\overline{s_{12}r_{21}}r_{12}s_{12}+\left(\overline{s_{12}}\right)^2 {s_{12}}^2$,

$G_1=-s_{12}r_{12}\overline{s_{11}s_{22}}+s_{12}r_{12}\left(\overline{s_{12}}\right)^2+s_{22}r_{11}\overline{s_{11}s_{22}}-s_{22}r_{11}\left(\overline{s_{12}}\right)^2-\overline{r_{12}s_{12}}r_{11}r_{22}+\overline{r_{12}s_{12}}r_{12}r_{21}+\overline{r_{22}s_{11}}r_{11}r_{22}-\overline{r_{22}s_{11}}r_{12}r_{21}$,

$G_0=s_{11}r_{12}\overline{s_{11}s_{22}}-s_{11}r_{12}\left(\overline{s_{12}}\right)^2-s_{12}r_{11}\overline{s_{11}s_{22}}+s_{12}r_{11}\left(\overline{s_{12}}\right)^2+\overline{r_{11}s_{12}}r_{11}r_{22}-\overline{r_{11}s_{12}}r_{12}r_{21}-\overline{r_{21}s_{11}}r_{11}r_{22}+\overline{r_{21}s_{11}}r_{12}r_{21}$,

$G_{-1}=s_{11}r_{22}\overline{s_{11}s_{22}}-s_{11}r_{22}\left(\overline{s_{12}}\right)^2-s_{12}r_{21}\overline{s_{11}s_{22}}+s_{12}r_{21}\left(\overline{s_{12}}\right)^2+\overline{r_{11}s_{22}}r_{11}r_{22}-\overline{r_{11}s_{22}}r_{12}r_{21}-\overline{r_{21}s_{12}}r_{11}r_{22}+\overline{r_{21}s_{12}}r_{12}r_{21}$.

APPENDIX C: The functions D_1, G_1, G_0 and G_{-1} in Eqs.(25)-(27)

$D_1=57-40\sqrt{2}-118\sqrt{2}\eta-134\sqrt{2}\eta^2-64\sqrt{2}\eta^3+20\sqrt{2}\eta^5+8\,\eta^6\sqrt{2}+4\,\eta^4\sqrt{2}-176\sqrt{2}\tau^2+256\,\tau^4\sqrt{2}+4096\,\tau^6\sqrt{2}+800\,\tau^2\eta^2+2304\,\tau^4\eta^2+704\,\eta\,\tau^2+640\,\tau^2\eta^3+2560\,\tau^4\eta+288\,\tau^2\eta^4-256\,\tau^2\sqrt{2}\eta+1280\,\tau^4\sqrt{2}\eta+192\,\tau^2\sqrt{2}\eta^4+64\,\tau^2\sqrt{2}\eta^2+1536\,\tau^4\sqrt{2}\eta^2+320\,\tau^2\sqrt{2}\eta^3+172\,\eta+214\,\eta^2+82\,\eta^4+152\,\eta^3+40\,\eta^5+12\,\eta^6+304\,\tau^2+1152\,\tau^4+6144\,\tau^6$,

$G_1=-133-110\,i\sqrt{2}\tau+512\,i\tau^5+928\,i\tau^3+376\,i\eta\,\tau+94\sqrt{2}+375\sqrt{2}\eta+618\sqrt{2}\eta^2+541\sqrt{2}\eta^3+78\sqrt{2}\eta^5+12\,\eta^6\sqrt{2}+270\,\eta^4\sqrt{2}+992\sqrt{2}\tau^2+3456\,\tau^4\sqrt{2}+6144\,\tau^6\sqrt{2}-3520\,\tau^2\eta^2-768\,\tau^4\eta^2-3680\,\eta\,\tau^2-1344\,\tau^2\eta^3-5376\,\tau^4\eta-96\,\tau^2\eta^4+2600\,\tau^2\sqrt{2}\eta+4992\,\tau^4\sqrt{2}\eta+288\,\tau^2\sqrt{2}\eta^4+2592\,\tau^2\sqrt{2}\eta^2+2304\,\tau^4\sqrt{2}\eta^2+1248\,\tau^2\sqrt{2}\eta^3-531\,\eta-876\,\eta^2-366\,\eta^4-764\,\eta^3-84\,\eta^5-4\,\eta^6-1408\,\tau^2-4736\,\tau^4-2048\,\tau^6+340\,i\tau\,\eta^2+8\,i\eta^4\tau+128\,i\tau^3\eta^2+128\,i\eta^3\tau+1024\,i\eta\,\tau^3-240\,i\tau\sqrt{2}\eta^2-24\,i\eta^4\sqrt{2}\tau-264\,i\sqrt{2}\eta\,\tau-832\,i\tau^3\sqrt{2}\eta-104\,i\sqrt{2}\eta^3\tau-384\,i\tau^3\sqrt{2}\eta^2+156\,i\tau-640\,i\sqrt{2}\tau^3-1536\,i\tau^5\sqrt{2}$,

$G_0=780+16644\,\eta^3-3252\sqrt{2}\eta-8296\sqrt{2}\eta^2-12000\sqrt{2}\eta^3-10840\,\eta^4\sqrt{2}-6372\sqrt{2}\eta^5-2472\,\eta^6\sqrt{2}-616\,\eta^7\sqrt{2}-80\,\eta^8\sqrt{2}-32768\,\tau^6\eta^2+139264\,\tau^6\eta+6528\,\eta^5\tau^2-512\,\eta^6\tau^2+52224\,\tau^4\eta^3-6144\,\tau^4\eta^4-9920\sqrt{2}\tau^2-62976\,\tau^4\sqrt{2}-192512\,\tau^6\sqrt{2}-327680\,\tau^8\sqrt{2}+272\,\eta^7-16\,\eta^8-65536\,\tau^8+167936\,\tau^6+5000\,i\sqrt{2}\eta^2\tau+4800\,i\sqrt{2}\eta^3\tau+2720\,i\eta^4\sqrt{2}\tau+928\,i\sqrt{2}\eta^5\tau+160\,i\eta^6\sqrt{2}\tau+14848\,i\tau^3\eta^3\sqrt{2}+24064\,i\sqrt{2}\eta\,\tau^3+26624\,i\tau^3\eta^2\sqrt{2}+59392\,i\tau^5\eta\sqrt{2}+30720\,i\tau^5\eta^2\sqrt{2}+3840\,i\tau^3\eta^4\sqrt{2}+2800\,i\sqrt{2}\eta\,\tau+768\,i\tau^3\eta^4+6144\,i\tau^5\eta^2+32\,i\eta^6\tau+648\,i\sqrt{2}\tau+8768\,i\sqrt{2}\tau^3+38912\,i\tau^5\sqrt{2}+81920\,i\tau^7\sqrt{2}-552\sqrt{2}+7760\,\eta^5+83456\,\tau^4+16384\,i\tau^7-29696\,i\tau^3\eta-2704\,i\eta^4\tau-6016\,i\eta^3\tau-3904\,i\eta\,\tau-448\,i\eta^5\tau-7168\,i\tau^3\eta^3-6784\,i\eta^2\tau-25856\,i\tau^3\eta^2-28672\,i\tau^5\eta-912\,i\tau-11776\,i\tau^3-33792\,i\tau^5-14784\,\tau^2\eta^5\sqrt{2}-206336\,\tau^4\eta^2\sqrt{2}-30720\,\tau^4\eta^4\sqrt{2}-180480\,\tau^4\eta\sqrt{2}-2560\,\eta^6\sqrt{2}\tau^2-315392\,\tau^6\sqrt{2}\eta-163840\,\tau^6\sqrt{2}\eta^2-75648\,\tau^2\eta^2\sqrt{2}-42560\,\tau^2\eta^4\sqrt{2}-73536\,\tau^2\eta^3\sqrt{2}-118272\,\tau^4\eta^3\sqrt{2}-42368\sqrt{2}\eta\,\tau^2+4588\,\eta+11652\,\eta^2+100992\,\tau^2\eta^2+190976\,\tau^4\eta^2+58656\,\eta\,\tau^2+89088\,\tau^2\eta^3+216064\,\tau^4\eta+39872\,\tau^2\eta^4+14520\,\eta^4+2328\,\eta^6+13920\,\tau^2$,

$G_{-1}=-124+88\sqrt{2}-32\,i\eta^6\tau\sqrt{2}-10240\,i\eta\,\tau^5\sqrt{2}-160\,i\eta^5\tau\sqrt{2}-6144\,i\eta^2\tau^5\sqrt{2}-768\,i\eta^4\tau^3\sqrt{2}-2560\,i\eta^3\tau^3\sqrt{2}-4096\,i\eta^2\tau^3\sqrt{2}-416\,i\eta^4\tau\sqrt{2}-3584\,i\tau^3\sqrt{2}\eta-432\,i\eta\,\tau\sqrt{2}-704\,i\eta^3\tau\sqrt{2}-744\,i\eta^2\tau\sqrt{2}+30208\,\eta^2\tau^4\sqrt{2}-104\,i\sqrt{2}\tau-1024\,i\eta^3\tau^3+512\,\eta^6\tau^2\sqrt{2}-1344\,i\tau^3\sqrt{2}-6144\,i\sqrt{2}\tau^5-16384\,i\tau^7\sqrt{2}+10048\,\eta^3\tau^2\sqrt{2}+3072\,i\eta\,\tau^3+24832\,\tau^4\sqrt{2}\eta+6208\,\eta^4\tau^2\sqrt{2}+144\,i\eta^4\tau-32\,i\eta^6\tau-4096\,i\eta\,\tau^5-6144\,i\eta^2\tau^5-768\,i\eta^4\tau^3+6016\,\tau^2\sqrt{2}\eta+576\,i\eta\,\tau+32768\,\eta^2\tau^6\sqrt{2}+2496\,\eta^5\tau^2\sqrt{2}+640\,i\eta^3\tau+19968\,\eta^3\tau^4\sqrt{2}+53248\,\eta\,\tau^6\sqrt{2}+1280\,i\tau^3\eta^2+6144\,\eta^4\tau^4\sqrt{2}+896\,i\eta^2\tau-64\,i\eta^5\tau+10368\,\eta^2\tau^2\sqrt{2}+500\sqrt{2}\eta+1224\sqrt{2}\eta^2+1696\,\eta^3\sqrt{2}+868\,\eta^5\sqrt{2}+1480\,\eta^4\sqrt{2}+104\,\eta^7\sqrt{2}+360\,\eta^6\sqrt{2}+16\,\eta^8\sqrt{2}+1472\sqrt{2}\tau^2+8704\,\tau^4\sqrt{2}+28672\,\tau^6\sqrt{2}+1024\,i\tau^5+65536\,\tau^8\sqrt{2}-16384\,i\tau^7+144\,i\tau+1536\,i\tau^3-11904\,\eta^2\tau^2-7840\,\eta\,\tau^2-6656\,\eta^2\tau^4-1472\,\eta^4\tau^2-8192\,\eta^3\tau^2-19456\,\eta\,\tau^4+6144\,\eta^4\tau^4+32768\,\eta^2\tau^6+512\,\eta^6\tau^2+1152\,\eta^5\tau^2+9216\,\eta^3\tau^4+24576\,\eta\,\tau^6-700\,\eta-1684\,\eta^2-1720\,\eta^4-2228\,\eta^3-88\,\eta^6-720\,\eta^5+16\,\eta^8+48\,\eta^7-2016\,\tau^2-9728\,\tau^4-4096\,\tau^6+65536\,\tau^8$.
